# Effect of a Mobile App on Preoperative Patient Preparation for Major Ambulatory Surgery: Protocol for a Randomized Controlled Trial

**DOI:** 10.2196/10938

**Published:** 2019-01-16

**Authors:** Manuel Herrera-Usagre, Vicente Santana, Ramon Burgos-Pol, Juan Pedro Oliva, Eliazar Sabater, Maria Rita-Acosta, Miguel Angel Casado, Susana Cruces, Manuel Pacheco, Carlos Solorzano Perez

**Affiliations:** 1 Andalusian Agency for Healthcare Quality Sevilla Spain; 2 Department of Sociology Pablo de Olavide University Sevilla Spain; 3 Pharmacoeconomics & Outcomes Research Iberia Paseo de Joaquín Rodrigo 4-I 28224 Pozuelo de Alarcón, Madrid Spain; 4 Hospital de Alta Resolución de Utrera, APS Bajo Guadalquivir Utrera, Sevilla Spain; 5 Hospital de Alta Resolución Sierra Norte, APS Bajo Guadalquivir Constantina, Sevilla Spain

**Keywords:** ambulatory surgical procedures, cost-benefit analysis, mobile phone, patient compliance, patient safety, preoperative care, telemedicine

## Abstract

**Background:**

Inadequate preoperative patient preparation causes organizational, economic, and emotional problems to patients and professionals. In Spain, no current evidence is available on either the rate of compliance or the impact of good compliance with preoperative recommendations by patients in the ambulatory setting. However, it is known that around 25% of surgical cancellations in the major ambulatory surgery (MAS) are due to poor compliance with these recommendations and, therefore, avoidable. Introducing innovative tools based on mobile health (mHealth) apps may help patients meet the preoperative recommendations and, consequently, reduce the rate of cancellations in the ambulatory setting.

**Objective:**

The objective of this study was to evaluate the effectiveness of the Listeo+ mHealth app as a tool for improving compliance with preoperative recommendations in MAS versus standard of care (SOC).

**Methods:**

A multicenter, randomized, open-label clinical trial that compares SOC with the additional use of Listeo+, a specific mHealth app for MAS preoperative patient monitoring, is being conducted. The study will include patients aged ≥18 years with surgical indication for MAS who meet the necessary technological and connectivity requirements. Patients in the control group will receive written preoperative recommendations, while those in the intervention group will additionally use the Listeo+ mHealth app. There will be a competitive recruitment of 790 patients during 6 months in 4 hospitals in Andalusia (Spain) that belong to the National Health System. The primary efficacy outcome is the level of compliance with preoperative recommendations. Secondary outcomes include the rate of cancellations, associated resource consumption, and perceived usability and utility with Listeo+ by participants of the intervention group. Simple randomization 1:1 procedure will be used to allocate patients to each study group.

**Results:**

The technological development of Listeo+ and the integration and interoperability of information systems was completed in September 2017. Subsequently, simulation tests were performed with Listeo+, and a pilot study was initiated with real patients that concluded successfully in October 2017. Patient recruitment began in December 2017 in the 4 participating centers. After an intermediate analysis performed 10 months after the start of the recruitment phase, the data collection and cleaning phases are estimated to be completed in April 2019, and the analysis with the final results will be conducted in July 2019.

**Conclusions:**

Progress in the integration and interoperability of information systems represents a major step forward in the field of mHealth. The app will allow health professionals to monitor in real-time patients’ preparation and critical preoperative recommendations fulfillment. We expect a reduction in avoidable preoperative cancellations due to a lack of or a poor patient preparation. Self-assessed Web-based questionnaires and focus group will provide important information about the perceived usability and utility of Listeo+ app among patients and health care professionals.

**International Registered Report Identifier (IRRID):**

DERR1-10.2196/10938

## Introduction

Providing quality care in different phases of the surgical process has become an important challenge for health systems because of the increase in the care burden, the increase in the complexity of surgical procedures, and increasingly demanding attention focused on patients’ preferences [1]. Throughout the surgical process, different factors (organizational, relative to patients’ clinical condition, or medical) can lead to surgery cancellations or surgical delay [2-4]. The implications of surgery cancellations can be analyzed from the perspectives of health management and patient safety, as their effects on health resource consumption can be considered adverse events (AEs) that require control and monitoring [5-7]. One major cause is the inadequate preoperative patient preparation because the safety guarantees for the intervention are not met [8,9]; this affects both the quality of the surgical procedure and the consumption of hospital resources as a result of the increase in hospital stay and consumption of medicines [1].

Major ambulatory surgery (MAS) is characterized by short-term postoperative care and does not require hospital admission; it has greatly increased in developed countries in recent decades [10]. In Spain, it represents 62.5% of the total number of surgeries performed by the National Health System (NHS) [11], which is one of the highest rates among Organisation for Economic Co-Operation and Development member countries [12]. Although the rate of cancellations in MAS is approximately 4% [13,14], lower than that reported in other countries where cancellations on the day of surgery oscillate between 5% and 40% [10,15], a quarter (27%) of those cancellations are because of poor compliance with preoperative recommendations and are, therefore, avoidable [13,16]. Conversely, inadequate preoperative patient preparation for MAS is also considered one of the main causes of patient no-shows on the day of surgery [17], which is likely due to patient anxiety before surgery [18].

There are tools such as preassessment clinics (PACs) and the surgical safety checklist (SSC) that help minimize risks in the preoperative process by assessing patients’ anesthetic risk [19] or verifying compliance with essential surgical aspects from the beginning to the end of surgery [20]. Regarding these two elements, one of the initiatives to ensure that the requirements established in the preoperative assessment are met is to provide recommendations to patients so they can participate in their own care in aspects such as the use of medications (eg, anticoagulants and biologicals) and hygienic and dietetic measures. In this way, we hypothesize that involving patients in the preoperative care and promoting them to meet the specific recommendations can lead to avoiding risky situations and, consequently, surgery cancellations.

Currently, there is evidence of the benefits of PACs and the SSC on the reduction of postoperative complications in the ambulatory setting [21], preoperative anxiety [18], and cancellations for medical reasons (eg, inappropriate use of medication before surgery) [22,23].

Some experiences based on information and communication technologies, such as incorporating the SSC in digital form to the electronic health record (EHR) or sending short message service text messages as a reminder of health appointments, have made it possible to increase compliance to treatment and surgical recommendations, reduce cancellations, and avoid no-shows [1,24-27]. Currently, mobile devices (tablet, mobile phone, and wearable devices) have a very high degree of penetration in Spain [28], Andalusia in particular, with 70.9% of the population (some 4.8 million inhabitants) connecting to the internet through these devices [29]. Because of its characteristics, (eg, mobility, instant access, connectivity, and variety of functionalities), mobile health (mHealth) can influence patients’ attitudes and behaviors and facilitate the asynchronous information exchange between patients and health professionals [30]. Some mHealth-based interventions such as the use of mHealth apps, have proven effective in the management of chronic diseases (eg, diabetes, asthma, and hypertension) by improving clinical parameters, compliance, and reducing disease costs [31]. Despite their great potential, the few initiatives undertaken thus far in the ambulatory surgical setting have been limited to postoperative patient monitoring [32,33]. Thus, there is no available evidence of the effect of mHealth apps on compliance with preoperative recommendations and, consequently, on the reduction of surgical cancellations.

Listeo+ is a multifunctional mobile app that provides personalized information to surgical patients (date and time of surgery), adjusted to their clinical condition. In addition to sending reminders on critical aspects of the operation at different times, Listeo+ monitors compliance with preoperative recommendations by establishing a communication channel between patients and health care professionals, which facilitates intervention in the case of possible AEs.

The aim of this study is to evaluate the impact of Listeo+ as a complement to standard of care (SOC) in patient compliance with preoperative recommendations, surgery cancellations, and associated resource consumption in the ambulatory surgical setting, in a clinical context of real-world evidence, and to evaluate the user experience with the app (perceived usability and utility).

## Methods

### Study Design

A multicenter, randomized, and open-label controlled trial was planned to evaluate the Listeo+ mHealth app as a complement to SOC in patients undergoing MAS. The study protocol has two arms: patients who receive preoperative written recommendations (control group) and patients who use the Listeo+ mHealth app as a multifunctional tool to monitor personal recommendations from health professionals (intervention group). The study considers guidelines and recommendations of the Standard Protocol Items: Recommendations for Interventional Trials [34] and Consolidated Standards of Reporting Trials statements [35].

The study protocol has been approved in a peer-review process by the Spanish Ministry of Economy and Competitiveness in its Technological Projects in Health call on July 14, 2015 (application identification DTS15/00228) and by the Andalusian Regional Ministry of Health in its Health Research Projects call on July 15, 2015 (application identification PI-0447-2014).

### Study Setting

Four High-Resolution Hospital Centers belonging to the Public Health System of Andalusia as a part of the NHS hospital network are recruiting patients for this study (Multimedia Appendix 1). High-Resolution Hospital Centers encourage ambulatory surgery and short-term hospitalization using MAS; thus, they were considered suitable centers to evaluate the initiative. Participating Hospital reference population is about 187,957 inhabitants.

### Eligibility Criteria

#### Characteristics and Selection Criteria of Patients Undergoing Major Ambulatory Surgery

Patients participating in this study will be adults aged ≥18 years at the start of the study who will undergo MAS in the specialties of traumatology, orthopedic surgery, ophthalmology, or general surgery.

#### Inclusion Criteria

To participate in this study, participants should be autonomous or dependent on one or more caregivers to perform their preoperative preparation, with the necessary technological and connectivity resources (ie, to dispose a smartphone or tablet mobile device with an Android or iOS operating system with an internet connection and familiarity with mobile technologies).

Patients who are autonomous to perform their preoperative preparation and lack the technological requirements but have caregivers with the necessary technological and connectivity resources who can supervise their preoperative preparation may also be included in the study.

#### Exclusion Criteria

Patients with two scheduled operations during the same clinical episode or time period will be excluded from the study. Nonautonomous patients, whose caregivers cannot be located when personal preoperative recommendations (Multimedia Appendix 2) are provided, and patients of the intervention group who have not downloaded and registered on Listeo+ will also be excluded. To avoid the loss of patients, a rescue procedure will be used with patients who, within 7-14 days, have not registered on the Listeo+ app. A telephone call will be made urging them to register on the Listeo+ app. If a patient has not registered within 72 hours (3 days) after the rescue call has been made, he or she will be excluded from the study.

### Study Outcomes

#### Primary Outcomes

The primary outcome will be measured as the average percentage of patient compliance with preoperative recommendations (the number of recommendations met by surgical intervention). Compliance with type 2 recommendations will be checked at the point of anesthesia consultation, whereas types 1 and 3 at the point of patient reception and preparation the day of surgery.

#### Secondary Outcomes

The secondary outcomes include the rate of surgery cancellations (the absolute number of cancellations compared with the number of scheduled operations for each study group in the study period) and the associated consumption of hospital resources assessed by a cost analysis between the control and intervention groups, so only direct costs will be considered. To evaluate the user experience with the Listeo+ app in the intervention group, the perceived usability and utility of mHealth apps will be analyzed. The level of usability, defined as the extent to which Listeo+ is utilized by users to achieve specific objectives of mHealth apps [36], will be evaluated exclusively in patients, whereas the perceived utility of Listeo+ will be evaluated in health professionals using qualitative techniques.

Then, the absolute change (numerical difference over the two study groups) and relative change (percentage of variation among the intervention group over the SOC) will be determined to assess the impact of the intervention for the primary and secondary outcomes (except for variables related to user experience and the level of usability).

### Participants

**Figure 1:**
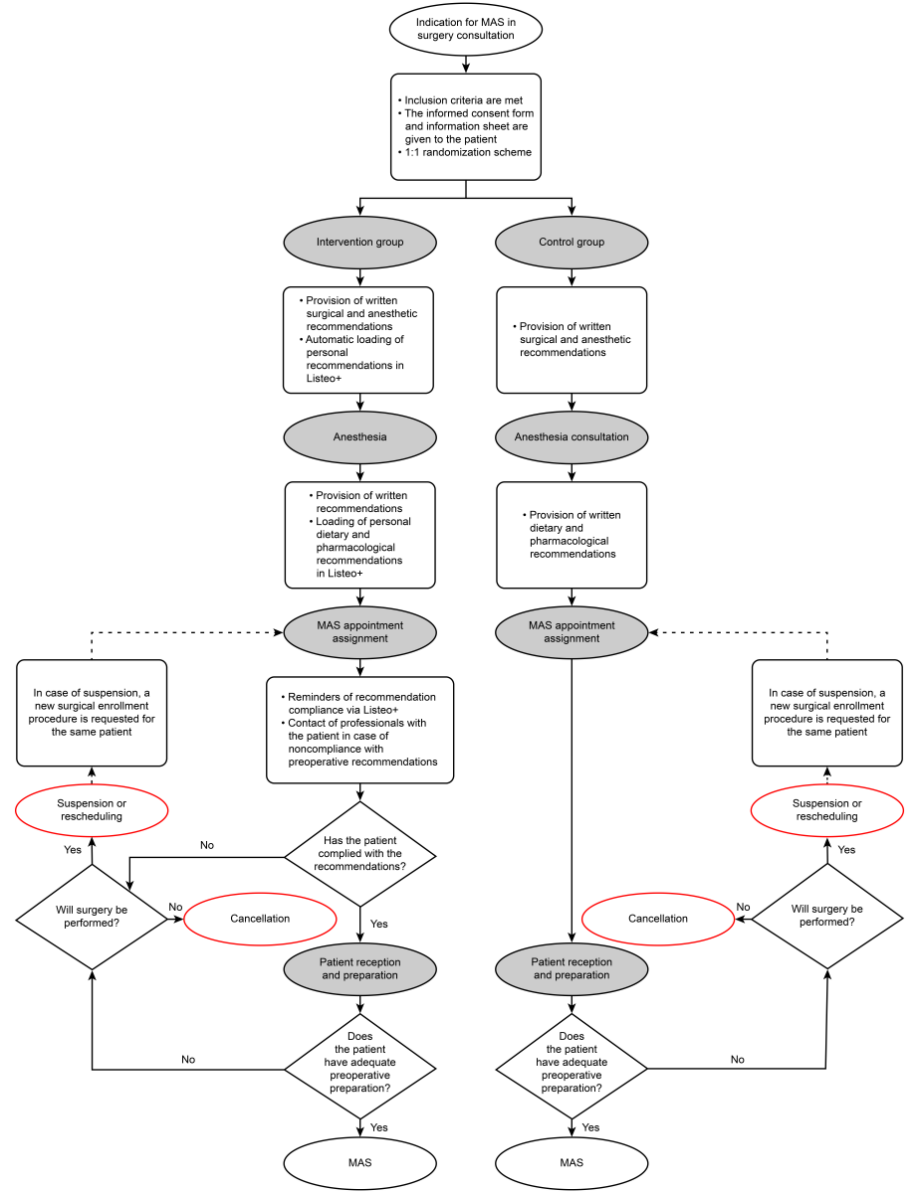
Patient flowchart. MAS: major ambulatory surgery.

Figure 1 presents the patient flow from the MAS assessment appointment to hospital discharge. This includes a first visit to surgery consultation, a second face-to-face anesthesia consultation (all patients except for ophthalmological patients with indication for topical anesthesia), and a third hospitalization visit to undergo MAS. Patients in the control group will follow the existing MAS patient assistance route in the centers, which consists of providing written recommendations. Participants are not going to pay for the app.

### Intervention

**Figure 2:**
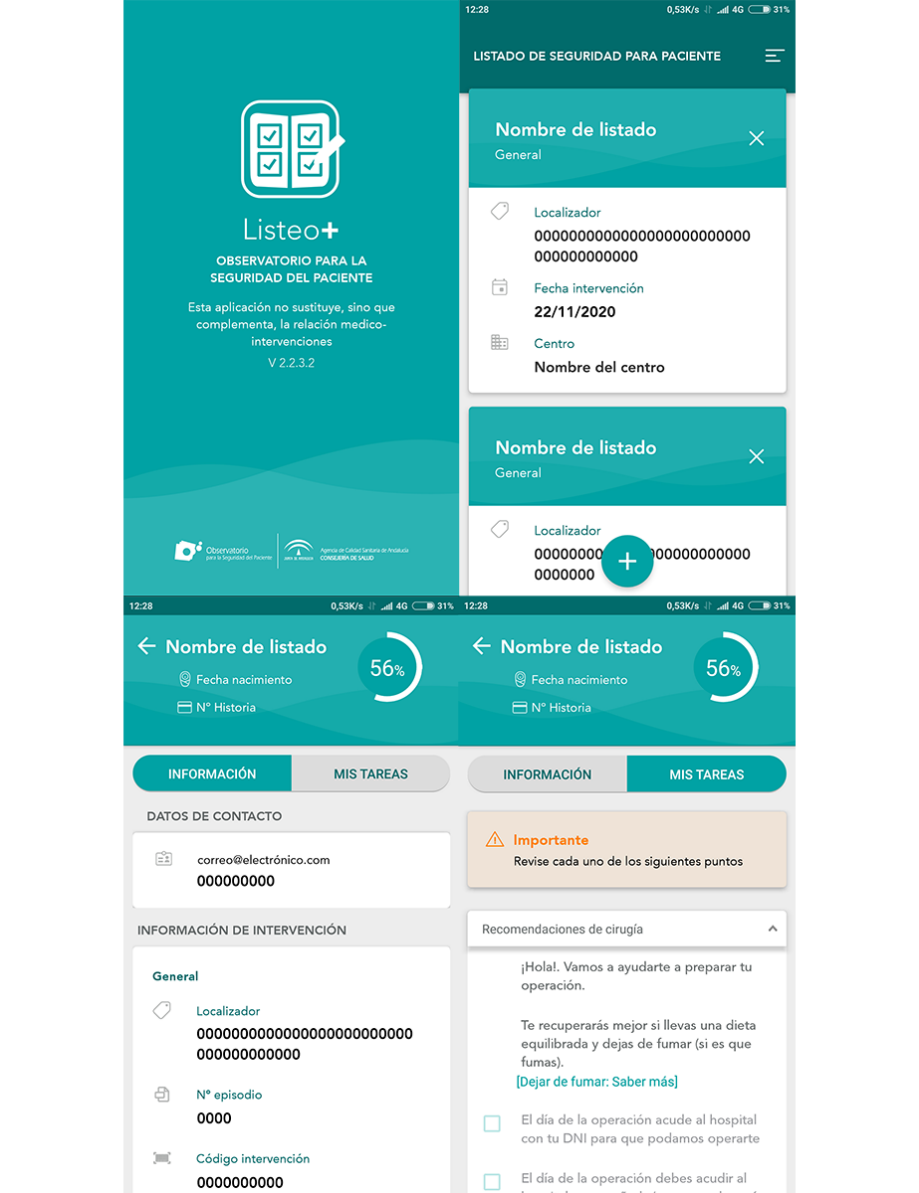
Screenshots of Listeo+ app.

**Figure 3:**
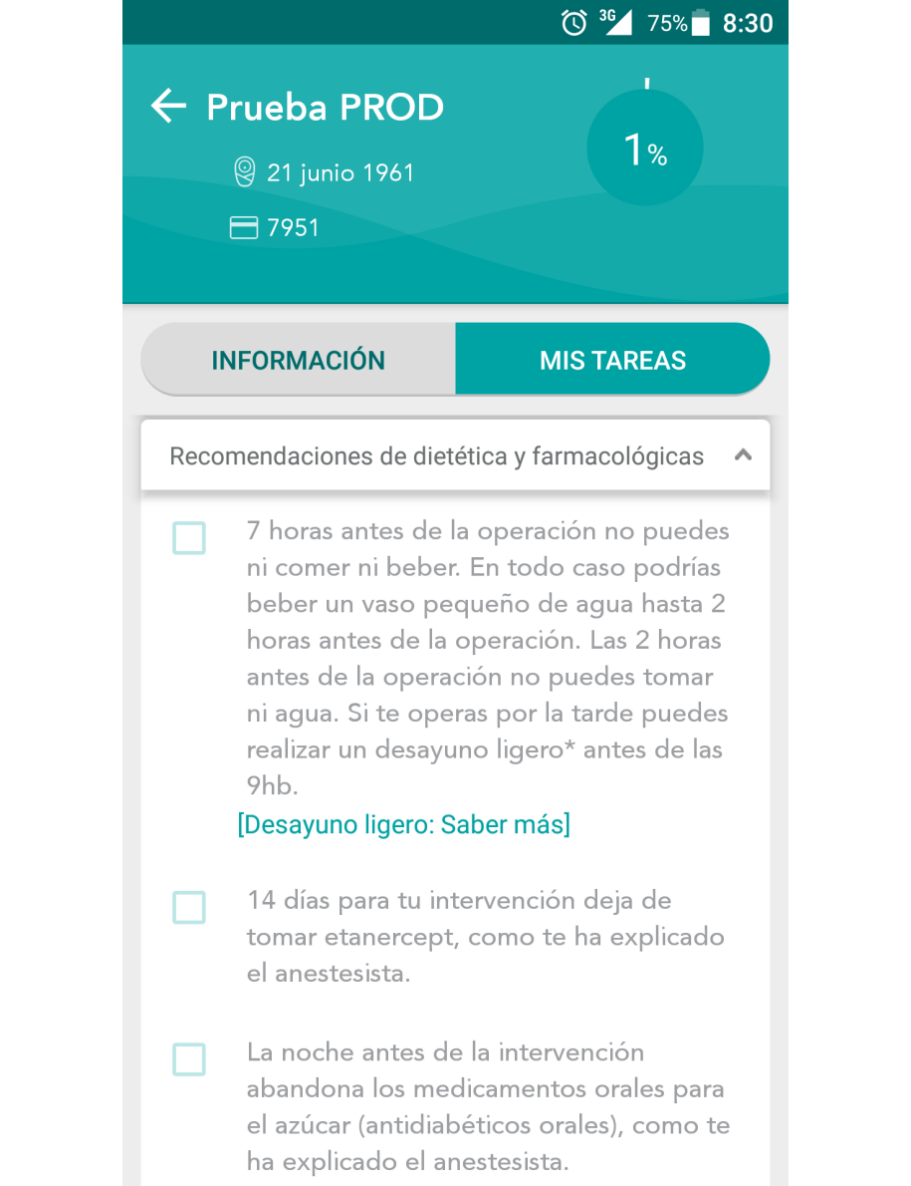
Screenshot of the to-do list for patients in the Listeo+ app.

Patients included in the intervention group will be provided with personal recommendations through the Listeo+ mobile app (Figures 2 and 3). These personal recommendations will also be printed and provided to the intervention group. Furthermore, the intervention group patients will be given access to download the mobile app through their mobile apps market (Google Play and Apple Store) using a link and quick response (QR)code.

**Figure 4:**
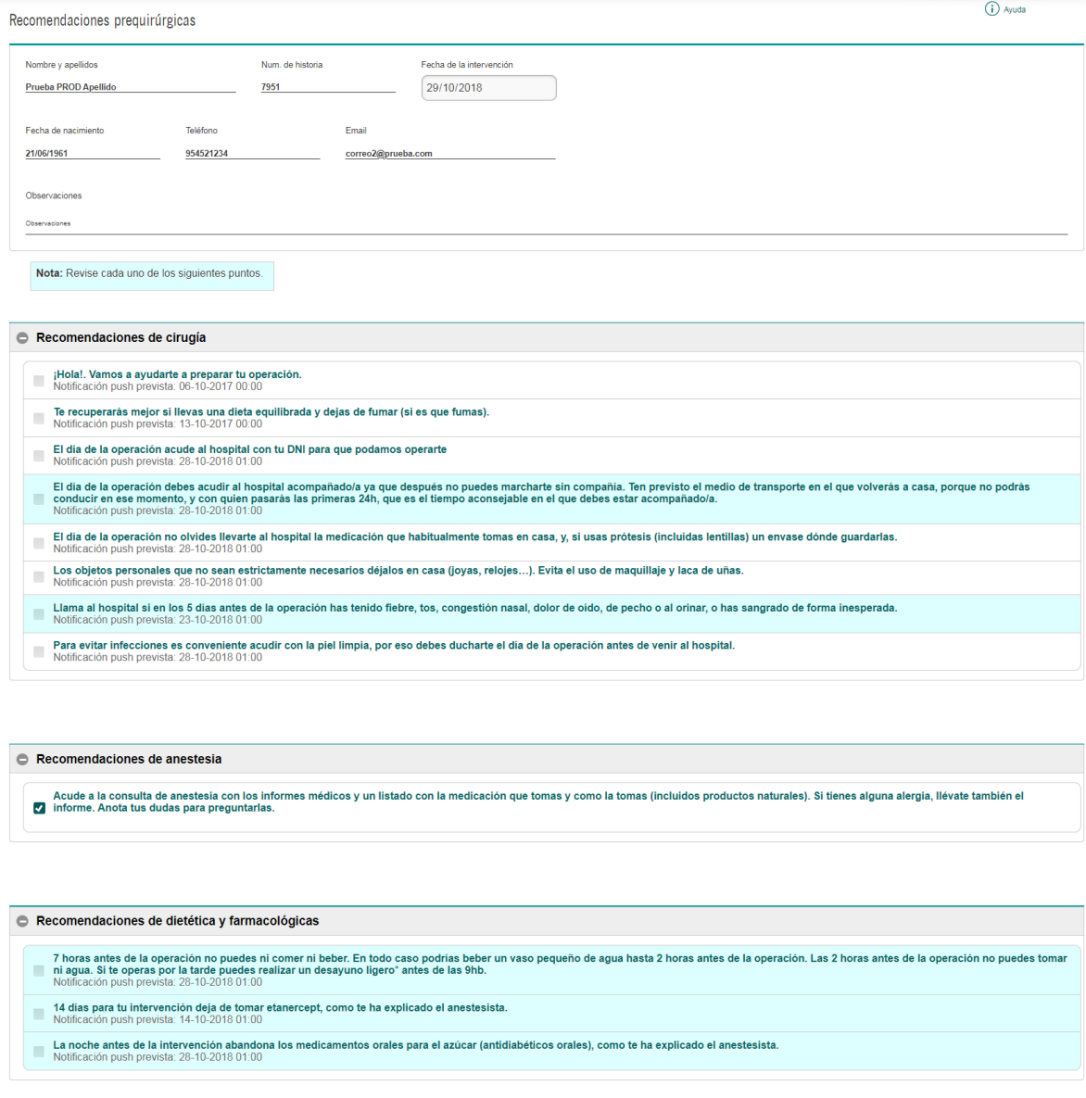
Screenshot of health care professionals’ Listeo+ module within the electronic health record platform.

Once the app is downloaded, patients will be able to use a personalized QR code included in their printed recommendations that will allow them to access their episode identification data and their personalized recommendations already set up by their anesthesiologist in the Web-based Listeo+ module integrated into the EHR platform of the Hospitals (Figure 4). Simultaneously, the EHR also sends all the necessary data for the app via Web service establishing the communication between the EHR and the app for the very first time.

Any interaction between app users and individualized recommendations will be immediately notified to the Listeo+ module and visualized in the EHR platform, which will automatically send notifications to the mobile app as well as emails to the designated health care professional of the participating center in case a critical recommendation (recommendations that may pose a risk to the patient or in which patient noncompliance may lead to the suspension, cancellation, or rescheduling of surgery as stated in Multimedia Appendix 2) is not marked. Additionally, health care professionals will be able to contact by phone to encourage patients to meet the recommendation or solve any potential problem. Notably, the nature of the incident will always be registered.

### Sample Size and Statistical Plan

The sample size required to estimate the main objective of this study was calculated as 395 patients per group for a total of 790 patients. This estimate was made assuming an alpha risk of .05, a beta risk of .2, and a difference in proportions between the groups of 10%. The analysis will be performed considering per intention-to-treat (ITT) and per protocol populations. The ITT population will include all randomized patients, whereas the per protocol population will include randomized patients who finally obtain an appointment to undergo MAS.

In addition, statistical analyses will be performed using the R software version 3.3.2 (R Foundation for Statistical Computing). For all analyses, an alpha risk of .05 will be assumed; therefore, to consider a statistically significant difference, the *P* value of the contrast statistic should be ≤.05. The statistical analysis planned *a priori* will consist of a descriptive analysis of the demographic and clinical characteristics of patients. For quantitative variables, the mean, SD, 95% CIs, variance, SE, 5% trimmed mean, median, minimum, and maximum will be calculated. For qualitative variables, frequency distributions with their respective percentages will be calculated. To determine whether there are differences in the level of compliance with surgical recommendations between the group with written recommendations and the group with written recommendations plus the app, Fisher's exact test will be performed.

To assess the influence of sociodemographic and clinical characteristics of patients in the level of compliance with preoperative recommendations, multivariate logistic regression will be performed. Furthermore, the reasons for the exclusion of the ITT population will be included.

### Allocation

Patients who meet the inclusion criteria and sign informed consent will be provided with an information sheet about the project and evaluated before participating in the study. To allocate patients to study groups, simple randomization 1:1 procedure will be used. To include patients in the study, each center will be provided with one randomization scheme generated by computer. Given the characteristics of the study, it is not possible to blind patients and professionals. Subsequently, we will collect sociodemographic data (age, sex, area of residence of patients, level of education of patients or caregivers using the app or patient or caregiver of the control group, occupation, marital status, and knowledge or handling of apps), clinical data (type of surgery, medical diagnosis [International Classification of Diseases, Ninth Revision], anesthetic evaluation, and medications taken), and functional situation by measuring disability (Barthel index).

#### Data Collection

All the study data will be collected through an electronic case report form (eCRF). To facilitate the completion of the eCRF, a specific module has been created and integrated into the EHR of the participating centers. The information that the researchers include in the eCRF will be exported to an anonymized database (without identifying patient data to ensure data confidentiality) for further analysis of the study data. The researchers will be responsible for creating a system that relates the numbers of the EHR (containing the eCRF data) with the anonymized code in the database where the data are exported and for maintaining the list of identification codes.

### Instruments

The Barthel index will be used to assess physical dependence and loss of autonomy of patients at the point of patient reception and preparation. Health care professionals will score patients based on whether they did or did not require physical assistance to perform daily activities, ranging from 0 (patient is dependent in all assessed activities) to 100 (patient is independent to perform the reflected activities) [37,38].

The criteria of the *American Society of Anesthesiologists* (ASA) will be used to evaluate the anesthetic risk and identify the clinical outcomes in patients at the point of anesthesia consultation. It scores patient’s overall health to describe 6 different levels (level I describes a normal healthy patient, whereas level VI describes a declared brain-dead patient whose organs are being removed for donor purposes) [39]. Only low-risk (ASA I and ASA II) patients are a candidate for MAS. Eventually, ASA III patients could benefit from MAS, after undergoing evaluation on individual basis for the risk-benefit balance of the ambulatory care (ie, ASA III without decompensation in the last 3 months) [10].

The information about mobile app usability by the intervention group will be collected through a modified version of the self-assessed mobile-based *Computer System Usability Questionnaire* [40]. Four focus groups—with 6-8 participants each—will be organized to collect qualitative data about the utility perceived by health professionals as well as intervention group patients’ experience with the app.

Both research tools will provide very insightful information that will eventually lead to changes in the Listeo+ functionality or content. In the event an incident occurs involving the cancellation, suspension, or rescheduling of surgery, the consumption of hospital resources will be recorded (consumption of medications, hospital stay, consumption of laboratory tests, diagnostic imaging, etc).

### Data Monitoring and Validation

Electronic monitoring of the completion of the eCRFs will be performed to detect missing information and possible data inconsistencies, thus, ensuring their quality. For this purpose, the researchers will be contacted during the patient recruitment phase, 3 and 6 months after the start of the project, using confidential information access codes. The inclusion of patients according to the established criteria (inclusion or exclusion criteria), the correct completion of the eCRFs, the signing and filing of the informed consent form of participating patients, and any other aspects required by the research team will be reviewed. The monitor will communicate to the corresponding research team the variables that must be reviewed in cases of lost or inconsistent data.

### Technological Development, Integration, and Interoperability

To ensure proper communication between mHealth app users (patients and health professionals), a process of integration of information systems and interoperability between Listeo+ and EHR has been developed. This process was planned in 4 phases: (1) codesign of the system and pilot; (2) integration and technical tests; (3) simulation and pilot testing; and (4) real environment testing.

### Ethical Aspects of the Study, Confidentiality, and Privacy

The study protocol has been evaluated and approved by the Regional Ethics Committee of Andalusia through the Biomedical Research Ethics Portal of Andalucía (PEIBA, for its acronym in Spanish). This study will be conducted in accordance with the principles of the latest version of the Declaration of Helsinki and will follow the Good Clinical Practice guidelines of Spain.

Written informed consent duly signed by all patients, legal representatives, or caregivers participating in this study will be collected before patient allocation to study groups. Data confidentiality will be protected under the Spanish law that ensures the protection of personal data (Organic Law on Protection of Personal Data, 15/1999, December 13). The researcher of each center will be responsible for keeping a study file containing patient identification and information, including the informed consent form signed by patients. Throughout the study, all related documents will be located in a secure area of the participating center. Any analysis derived from the study will be performed from an anonymized database; it will not contain any identification of patients or caregivers but only a numeric code, through which it will not be possible to reveal their identities. At the end of the study, the researcher will be responsible for preserving the necessary documentation for at least 5 years.

## Results

Currently, this study is in the recruitment phase, which will end once 790 patients are included (395 for each arm). The data collection and cleaning phases are estimated to be completed in April 2019, and the analysis with the final results will be conducted in July 2019.

Previously, the technical aspects of interoperability between the hospital and the Listeo+ app backend (set of system components accessible only to the developers or platform administrators) were resolved successfully, defining an application programming interface for Web services. In December 2016, an eCRF was created that was fully integrated into the EHR of the participating centers.

Prior to the recruitment of patients, a pilot phase was conducted in January 2017 with the aim of identifying complications in the subsequent phases of recruitment and data analysis. During the pilot phase, face-to-face sessions were held in the hospitals with both health professionals and specialized information and communication technologies personnel. In these sessions, test runs were performed with several patients, verifying the effective communication between the systems and the usability of the new functionality integrated into the EHR. As a result, a telephone call was included in the protocol, at 7 and 14 days after the provision of recommendations during the anesthesiologist appointment, for the patients of the intervention group who had not downloaded their personal preoperative recommendations using the QR code. In addition, two new recommendations were added (see Multimedia Appendix 2: R1_18 and R3_24), and the wording of the recommendations was modified to facilitate patient understanding. Simultaneously, improvements were made in the design and functionality of Listeo+.

## Discussion

### Principal Findings

The introduction of new multifunctional technologies allows achieving different objectives in patient preparation, providing personalized information, and establishing an effective communication channel that facilitates patient monitoring by health professionals [30]. The evaluation of initiatives based on new technologies in the health sector is a fundamental element because of its subsequent adoption by the different stakeholders (patients, health professionals, and decision makers). According to the World Health Organization (WHO), the lack of evidence on the effectiveness and economic impact of mHealth-based interventions is one of the most important barriers for implementing these programs within the framework of the European Union [41]. In this sense, it is necessary to perform initiatives aimed at generating evidence on the effect of compliance with preoperative recommendations and their economic impact in MAS using mHealth apps, which evaluate their utility and efficiency in critical areas such as surgical patient safety. Taking into account the increasingly important role of citizens and patients in health systems, the possibility of having information about user experience (perceived usability and utility) makes it possible to evaluate the suitability of these tools in a real clinical setting.

### Relevance of the Study

Improvements in systems integration and interoperability could have great relevance. Currently, it has been possible to incorporate into the EHR of the participating centers a generator of preoperative recommendation lists that allows selecting the information according to individual patient characteristics. In addition, the structure of information systems for data exchange has been modified from the users’ mobile device using Listeo+ and the EHR in these centers. The learning process and the improvement in systems integration and interoperability can be used for other initiatives within the framework of mHealth apps in Andalusia, a region with a favorable environment for the development of initiatives based on new technologies and, by extension, to the rest of the NHS.

Furthermore, the interest of the study lies in the increase in MAS and the adoption of mobile devices and acceptance of mHealth apps by the population. Thus, the number of MAS operations in developed countries has continued to increase in recent years. In 2015, in Spain, 1,632,824 MAS operations were performed, corresponding to an increase of 4.2% from the period of 2010-2015 [11]. In addition, it highlights that the penetration level of smartphones is also increasing, even among the elderly, reducing the generation gap [42]. Finally, data from a local survey on the use of mHealth apps show that 73.8% of patients would use them if recommended by their doctor, which suggests a high level of acceptance of mHealth apps by the population [43].

### Limitations

This study has some limitations related to the design of the intervention and the methodology used. First, it is a randomized clinical trial with an *open-label* design. Although not blinding patients and professionals could lead to potential bias in the interpretation of results, this type of design is widely accepted in complex nonpharmacological interventions (eg, surgery and medical devices) [44] in which masking cannot be applied. Second, Listeo+ has been evaluated as a complement to SOC (written recommendations). In this sense, other published clinical trials based on MAS and Patient Support Programs also use this methodological approach where intervention is assumed as a complement to SOC [45,46].

### Conclusions

In line with WHO guidelines, mHealth apps help search for new formulas that support patient safety by involving them in the care process and making them responsible for their own safety. Listeo+ mobile app will allow health professionals to monitor in real-time patients’ preparation and critical preoperative recommendation fulfillment. The achievements obtained in the integration and interoperability of information systems prior to recruitment are considered a fundamental advancement in the development of strategies for mHealth app-based solutions. As a result, a reduction in avoidable preoperative cancellations due to a lack of or a poor patient preparation is expected, and self-assessed Web-based questionnaires and focus group will provide important information about the perceived usability and utility of Listeo+ app among patients and health care professionals.

## Appendix

Multimedia Appendix 1 Listeo+ promotional video.

Multimedia Appendix 2 MAS preoperative recommendations in Listeo+.

Multimedia Appendix 3 CONSORT-EHEALTH checklist (V 1.6.1).

Multimedia Appendix 4 Peer-review report and trial registration from the national funding agency.

Multimedia Appendix 5 Peer-review report and trial registration from the regional funding agency.

## Abbreviations

ASA: American Society of Anesthesiologists
HER: electronic health record
ITT: intention-to-treat
MAS: major ambulatory surgery
NHS: National Health System
QR: quick response
SOC: standard of care
SSC: surgical safety checklist
WHO: World Health Organization
