# Supplementary material for: Effect of a Mobile App on Preoperative Patient Preparation for Major Ambulatory Surgery: Protocol for a Randomized Controlled Trial
Source: JMIR Res Protoc. 2019 Jan 16;8(1):e10938. doi: 10.2196/10938 (PMC6352007; doi:10.2196/10938)
Supplement: Multimedia Appendix 2 [file resprot_v8i1e10938_app2.pdf]

Table 1. MAS preoperative recommendations in Listeo+.

	#	Recommendation	General/selectable	Notification time
Type 1	**R1_01**	Hello! We are going to help you prepare for your operation.	**General**	7 days after entering the RSD
	**R1_02**	You will recover better if you have a balanced diet and quit smoking (if you smoke)[a].	**General**	7 days after entering the RSD
	**R1_03**	On the day of surgery, go to the hospital with your ID so we can perform the surgery.	**General**	24 hours before surgery
	**R1_04**	On the day of surgery, you must go to the hospital accompanied by someone else since you cannot leave afterwards without company. Plan the means of transport by which you will return home because you will not be able to drive at that time, and with whom you will spend the first 24 hours, which is the advisable time during which you must be accompanied.	**General**	24 hours before surgery
	**R1_05**	On the day of surgery, do not forget to take the medication you usually take at home to the hospital, and, if you use prosthesis (including contact lenses), a container to store them.	**General**	24 hours before surgery
	**R1_06**	Leave personal items that are not strictly necessary at home (jewelry, watches...). Do not wear makeup or nail polish.	**General**	24 hours before surgery
	**R1_07**	Call the hospital if you have a fever, cough, nasal congestion, earache, chest pain, pain urinating, or unexpected bleeding five days before surgery.	**General**	6 days before surgery
	**R1_08**	To avoid infections, it is convenient to attend the hospital with clean skin, so you should shower on the day of surgery before coming to the hospital.	**General**	24 hours before surgery
	**R1_09**	On the day of surgery, before showering, trim the hair of the breast to be operated on and the closest armpit, to approximately one millimeter. For this, you can use scissors or an electric razor.	**Selectable** *(male breast surgery)*	24 hours before surgery
	**R1_10**	On the day of surgery, before showering, trim leg hair to approximately one millimeter. For this, you can use scissors or an electric razor.	**Selectable** *(varicose sclerosis*	24 hours before surgery

			in males)	
	**R1_11**	On the day of surgery, before showering, trim abdomen hair to approximately one millimeter. For this, you can use scissors or an electric razor.	**Selectable** *(open supraumbilical eventration in males/umbilical hernias in males)*	24 hours before surgery
	**R1_12**	On the day of surgery, before showering, trim the hair from the anus to the buttocks (included) to approximately one millimeter. For this, you can use scissors or an electric razor.	**Selectable** (coccygeal cyst)	24 hours before surgery
	**R1_13**	On the day of surgery, it is important that the underwear you bring to the hospital to use after discharge is elastic and tight.	**Selectable** *(inguinal hernia)*	24 hours before surgery
	**R1_14**	On the day of surgery, it is important that the bra that you take to the hospital to use after discharge is elastic and tight (sports type).	**Selectable** *(breast surgery)*	24 hours before surgery
	**R1_15**	Remember that you can eat. You do not need to fast prior to your surgery.	**Selectable** *(local topical anesthesia)*	24 hours before surgery
	**R1_16**	Remember that it is not advisable to consume dairy products, fried foods, eggs, and fatty foods because they can worsen symptoms.	**Selectable** *(cholecystectomy)*	From the time of entering the RSD
	**R1_17**	For your surgery, it is very important that you do not attend the hospital wearing makeup.	**Selectable** *(ophthalmology)*	24 hours before surgery
	**R1_18**	Do not forget to administer the laxative (250 cc) the night before surgery, as we explained during the consultation.	**Selectable** *(Proctologic surgery)*	24 hours before surgery
Type 2	**R2_01**	Go to the anesthesia consultation with the medical reports and a list of the medications you take and dosing schedule (including natural products). If you have any allergies, be sure to report them as well. Write down your doubts to ask them.	**General**	24 hours before the anesthesia consultation
Type 3	**R3_01**	You cannot eat or drink seven hours before surgery. In any case, you can drink a small glass of water until two hours before surgery. You cannot drink water two hours before surgery. If your surgery is scheduled in the afternoon, you can have a light* breakfast before 9:00[b]**.**	**Selectable**	24 hours before surgery

	**R3_02**	Three days before surgery, stop taking acenocoumarol (Sintrom®) and replace it with heparin if the hematologist or anesthetist have directed you.	**Selectable**	4 days before surgery
	**R3_03**	Four days before surgery, stop taking warfarin (Aldocumar®) and replace it with heparin if the hematologist or anesthesiologist have directed you.	**Selectable**	5 days before surgery
	**R3_04**	Two days before surgery, stop taking cilostazol (Pletal® or Ekistol®) and replace it with 100 mg of acetylsalicylic acid (Aspirin® 100 mg), as the anesthetist has directed you.	**Selectable**	3 days before surgery
	**R3_05**	Seven days before surgery stop taking prasugrel (Efient®, and replace it with 100 mg of acetylsalicylic acid (Aspirin® 100 mg), as the anesthesiologist has directed you.	**Selectable**	8 days before surgery
	**R3_06**	Five days before surgery, stop taking ticagrelor (Brilique® or Possia®) and replace it with 100 mg of acetylsalicylic acid (Aspirin® 100 mg), as the anesthetist has directed you.	**Selectable**	6 days before surgery
	**R3_07**	Two days before surgery, stop taking apixaban (Eliquis®), as the anesthetist has directed you.	**Selectable**	3 days before surgery
	**R3_08**	Fourteen days before surgery, stop taking etanercept, as the anesthetist has directed you.	**Selectable**	15 days before surgery
	**R3_09**	Seven days before surgery, stop taking leflunomide, as the anesthetist has directed you.	**Selectable**	8 days before surgery
	**R3_10**	Six weeks before surgery, stop taking infliximab, golimumab, or certolizumab, as the anesthetist has directed you.	**Selectable**	7 days before surgery
	**R3_11**	Three weeks before surgery, stop taking adalimumab, as the anesthetist has directed you.	**Selectable**	22 days before surgery
	**R3_12**	Four weeks before surgery, you should stop oral contraceptives and use alternative contraceptive methods for up to a week after the operation.	**Selectable**	29 days before surgery
	**R3_13**	Ten days before surgery, stop taking meloxicam or piroxicam and replace them with	**Selectable**	11 days before surgery

		another analgesic, as the anesthetist has directed you.		
	**R3_14**	Two days before surgery, stop taking ibuprofen, naproxen, ketoprofen, or indomethacin and replace it with another analgesic, as the anesthetist has directed you.	**Selectable**	3 days before surgery
	**R3_15**	Seven days before surgery, stop taking the medication of medicinal plants containing garlic, Gingko biloba, Ginseng, St. John's wort, Hypericum, or Ephedra, as the anesthetist has directed you.	**Selectable**	8 days before surgery
	**R3_16**	The night before surgery, stop taking oral medications for high blood sugar (oral antidiabetics), as the anesthetist has directed you.	**Selectable**	24 before surgery
	**R3_17**	The night before surgery, reduce the slow insulin dose by half and do not administer any on the day of surgery, as the anesthetist has directed you.	**Selectable**	24 before surgery
	**R3_18**	On the day of surgery, do not forget to take your usual medication, except for those that we have told you to stop.	**Selectable**	24 before surgery
	**R3_19**	Two days before surgery, stop taking dabigatran (Pradaxa®), as the anesthetist has directed you.	**Selectable**	3 days before surgery
	**R3_20**	Two days before surgery, stop taking rivaroxaban (Xarelto®), as the anesthetist has directed you.	**Selectable**	3 days before surgery
	**R3_21**	Seven days before surgery, stop taking 300 mg of acetylsalicylic acid (Aspirin® 300 mg) and replace it with 100 mg of acetylsalicylic acid (Aspirin® 100 mg), as the anesthetist has directed you.	**Selectable**	8 days before surgery
	**R3_22**	Seven days before surgery, stop taking ticlopidine (Ticlodone® or Tiklid®) and replace it with 100 mg of acetylsalicylic acid (Aspirin® 100 mg), as the anesthetist has directed you.	**Selectable**	8 days before surgery
	**R3_23**	Seven days before surgery, stop taking clopidogrel (Plavix®) and replace it with 100 mg of acetylsalicylic acid (Aspirin® 100 mg), as the anesthetist has directed you.	**Selectable**	8 days before surgery
	**R3_24**	The anesthetist has established specific recommendations for your case. Check the anesthesia consultation report and make sure you follow the recommendations.	**Selectable**	8 days before surgery

[a]This recommendation is accompanied by additional information on how to quit smoking ("Know more").
[b]This recommendation is accompanied by additional information on what constitutes a light breakfast ("Know more").

⚠: Recommendations that may pose a risk to the patient or in which patient non-compliance may lead to the suspension, cancellation, or rescheduling of surgery.
RSD: Registry of Surgical Demand;
Type 1: Surgical recommendations; Type 2: Anesthesia recommendations; Type 3: Dietary and pharmacological recommendations
