# Supplementary material for: Effect of a Mobile App on Preoperative Patient Preparation for Major Ambulatory Surgery: Protocol for a Randomized Controlled Trial
Source: JMIR Res Protoc. 2019 Jan 16;8(1):e10938. doi: 10.2196/10938 (PMC6352007; doi:10.2196/10938)
Supplement: Multimedia Appendix 3 [file resprot_v8i1e10938_app3.pdf]

# CONSORT-EHEALTH (V 1.6.1) - Submission/Publication Form

The CONSORT-EHEALTH checklist is intended for authors of randomized trials evaluating web-based and Internet-based applications/interventions, including mobile interventions, electronic games (incl multiplayer games), social media, certain telehealth applications, and other interactive and/or networked electronic applications. Some of the items (e.g. all subitems under item 5 - description of the intervention) may also be applicable for other study designs.

The goal of the CONSORT EHEALTH checklist and guideline is to be
a) a guide for reporting for authors of RCTs,
b) to form a basis for appraisal of an ehealth trial (in terms of validity)

CONSORT-EHEALTH items/subitems are MANDATORY reporting items for studies published in the Journal of Medical Internet Research and other journals / scientific societies endorsing the checklist.

Items numbered 1., 2., 3., 4a., 4b etc are original CONSORT or CONSORT-NPT (non-pharmacologic treatment) items.
Items with Roman numerals (i., ii, iii, iv etc.) are CONSORT-EHEALTH extensions/clarifications.

As the CONSORT-EHEALTH checklist is still considered in a formative stage, we would ask that you also RATE ON A SCALE OF 1-5 how important/useful you feel each item is FOR THE PURPOSE OF THE CHECKLIST and reporting guideline (optional).

Mandatory reporting items are marked with a red *.
In the textboxes, either copy & paste the relevant sections from your manuscript into this form - please include any quotes from your manuscript in QUOTATION MARKS,
or answer directly by providing additional information not in the manuscript, or elaborating on why the item was not relevant for this study.

YOUR ANSWERS WILL BE PUBLISHED AS A SUPPLEMENTARY FILE TO YOUR PUBLICATION IN JMIR AND ARE CONSIDERED PART OF YOUR PUBLICATION (IF ACCEPTED).
Please fill in these questions diligently. Information will not be copyedited, so please use proper spelling and grammar, use correct capitalization, and avoid abbreviations.

DO NOT FORGET TO SAVE AS PDF _AND_ CLICK THE SUBMIT BUTTON SO YOUR ANSWERS ARE IN OUR DATABASE !!!

Citation Suggestion (if you append the pdf as Appendix we suggest to cite this paper in the caption):
Eysenbach G, CONSORT-EHEALTH Group
CONSORT-EHEALTH: Improving and Standardizing Evaluation Reports of Web-based and Mobile Health Interventions
J Med Internet Res 2011;13(4):e126
URL: http://www.jmir.org/2011/4/e126/
doi: 10.2196/jmir.1923
PMID: 22209829

*Obligatorio

## Your name *

First Last

Manuel Herrera-Usagre

## Primary Affiliation (short), City, Country *

University of Toronto, Toronto, Canada

Andalusian Agency for Healthcare Qualit

## Your e-mail address *

abc@gmail.com

manuel.herrera.usagre@juntadeandaluc

## Title of your manuscript *

Provide the (draft) title of your manuscript.

Effect of a Mobile Application on Preoperative Patient Preparation for Major Ambulatory Surgery: Protocol for a Randomized Crontrolled Trial

## Name of your App/Software/Intervention *

If there is a short and a long/alternate name, write the short name first and add the long name in brackets.

Listeo+

## Evaluated Version (if any)

e.g. "V1", "Release 2017-03-01", "Version 2.0.27913"

V.2.2.3.2

## Language(s) *

What language is the intervention/app in? If multiple languages are available, separate by comma (e.g. "English, French")

Spanish

## URL of your Intervention Website or App

e.g. a direct link to the mobile app on app in appstore (itunes, Google Play), or URL of the website. If the intervention is a DVD or hardware, you can also link to an Amazon page.

www.http://listeo.acsa.junta-andalucia.e

## URL of an image/screenshot (optional)

http://listeo.acsa.junta-andalucia.es/age

## Accessibility *

Can an enduser access the intervention presently?

- (●) access is free and open
- ( ) access only for special usergroups, not open
- ( ) access is open to everyone, but requires payment/subscription/in-app purchases
- ( ) app/intervention no longer accessible
- ( ) Otros:

## Primary Medical Indication/Disease/Condition *

e.g. "Stress", "Diabetes", or define the target group in brackets after the condition, e.g. "Autism (Parents of children with)", "Alzheimers (Informal Caregivers of)"

Patients undergoing Major Ambulatory S

## Primary Outcomes measured in trial *

comma-separated list of primary outcomes reported in the trial

Average percentage of patient complian

## Secondary/other outcomes

Are there any other outcomes the intervention is expected to affect?

Rate of surgery cancellations (absolute number of cancellations compared to the number of scheduled operations for each study group in the study period) and the associated consumption of hospital resources assessed by a cost analysis between the control and intervention group, so only direct costs will be considered. To evaluate the user experience with the Listeo+ application in the intervention group, the perceived usability and utility of mobile Health Apps will be analyzed.

## Recommended "Dose" *

What do the instructions for users say on how often the app should be used?

- ○ Approximately Daily
- ○ Approximately Weekly
- ○ Approximately Monthly
- ○ Approximately Yearly
- ◉ "as needed"
- ○ Otros:

## Approx. Percentage of Users (starters) still using the app as recommended after 3 months *

- (x) unknown / not evaluated
- ( ) 0-10%
- ( ) 11-20%
- ( ) 21-30%
- ( ) 31-40%
- ( ) 41-50%
- ( ) 51-60%
- ( ) 61-70%
- ( ) 71%-80%
- ( ) 81-90%
- ( ) 91-100%
- ( ) Otros:

## Overall, was the app/intervention effective? *

- ( ) yes: all primary outcomes were significantly better in intervention group vs control
- ( ) partly: SOME primary outcomes were significantly better in intervention group vs control
- ( ) no statistically significant difference between control and intervention
- ( ) potentially harmful: control was significantly better than intervention in one or more outcomes
- ( ) inconclusive: more research is needed
- (x) Otros: Still a study protocol

## Article Preparation Status/Stage *

At which stage in your article preparation are you currently (at the time you fill in this form)

- ○ not submitted yet - in early draft status
- ○ not submitted yet - in late draft status, just before submission
- ○ submitted to a journal but not reviewed yet
- ● submitted to a journal and after receiving initial reviewer comments
- ○ submitted to a journal and accepted, but not published yet
- ○ published
- ○ Otros:

## Journal *

If you already know where you will submit this paper (or if it is already submitted), please provide the journal name (if it is not JMIR, provide the journal name under "other")

- ○ not submitted yet / unclear where I will submit this
- ○ Journal of Medical Internet Research (JMIR)
- ○ JMIR mHealth and UHealth
- ○ JMIR Serious Games
- ○ JMIR Mental Health
- ○ JMIR Public Health
- ○ JMIR Formative Research
- ○ Other JMIR sister journal
- ● Otros: JMIR Research Protocols

## Is this a full powered effectiveness trial or a pilot/feasibility trial? *

- ● Pilot/feasibility
- ○ Fully powered

## Manuscript tracking number *

If this is a JMIR submission, please provide the manuscript tracking number under "other" (The ms tracking number can be found in the submission acknowledgement email, or when you login as author in JMIR. If the paper is already published in JMIR, then the ms tracking number is the four-digit number at the end of the DOI, to be found at the bottom of each published article in JMIR)

- ○ no ms number (yet) / not (yet) submitted to / published in JMIR
- ◉ Otros: doi:10.2196/10938

## TITLE AND ABSTRACT

## 1a) TITLE: Identification as a randomized trial in the title

## 1a) Does your paper address CONSORT item 1a? *

I.e does the title contain the phrase "Randomized Controlled Trial"? (if not, explain the reason under "other")

- ○ yes
- ◉ Otros: We rather use "Randomized Clinical Trial"

## 1a-i) Identify the mode of delivery in the title

Identify the mode of delivery. Preferably use “web-based” and/or “mobile” and/or “electronic game” in the title. Avoid ambiguous terms like “online”, “virtual”, “interactive”. Use “Internet-based” only if Intervention includes non-web-based Internet components (e.g. email), use “computer-based” or “electronic” only if offline products are used. Use “virtual” only in the context of “virtual reality” (3-D worlds). Use “online” only in the context of “online support groups”. Complement or substitute product names with broader terms for the class of products (such as “mobile” or “smart phone” instead of “iphone”), especially if the application runs on different platforms.

| | 1 | 2 | 3 | 4 | 5 | |
|---|---|---|---|---|---|---|
| subitem not at all important | ○ | ○ | ○ | ○ | ◉ | essential |

## Does your paper address subitem 1a-i? *

Copy and paste relevant sections from manuscript title (include quotes in quotation marks "like this" to indicate direct quotes from your manuscript), or elaborate on this item by providing additional information not in the ms, or briefly explain why the item is not applicable/relevant for your study

In the title we specified this is a Protocol for a "Mobile Application" Randomized Clinical Trial.
In the manuscript, We didn't use ambiguous terms like "online", "virtual" or "interactive". Instead, we use "mobile applicaton", "mobile app" or "web-based Listeo+ module integrated in the Hospitals EHR platform". Instead of using product names like "iphone" we have used "smartphone" or "tablet mobile device with an Android or iOS operating system"

## 1a-ii) Non-web-based components or important co-interventions in title

Mention non-web-based components or important co-interventions in title, if any (e.g., “with telephone support”).

| | 1 | 2 | 3 | 4 | 5 | |
|---|---|---|---|---|---|---|
| subitem not at all important | ○ | ◉ | ○ | ○ | ○ | essential |

## Does your paper address subitem 1a-ii?

Copy and paste relevant sections from manuscript title (include quotes in quotation marks "like this" to indicate direct quotes from your manuscript), or elaborate on this item by providing additional information not in the ms, or briefly explain why the item is not applicable/relevant for your study

We didn't use it in the title. However, we did mention it in the manuscript when we specify that [in order to] "avoid loss of patients, a rescue procedure will be used with those patients who, within a period of 7 to 14 days, have not registered on the Listeo+ app. A telephone call will be made urging them to register on the Listeo+ app."

## 1a-iii) Primary condition or target group in the title

Mention primary condition or target group in the title, if any (e.g., “for children with Type I Diabetes”)
Example: A Web-based and Mobile Intervention with Telephone Support for Children with Type I Diabetes: Randomized Controlled Trial

| | 1 | 2 | 3 | 4 | 5 | |
|---|---|---|---|---|---|---|
| subitem not at all important | ○ | ○ | ○ | ○ | ◉ | essential |

## Does your paper address subitem 1a-iii? *

Copy and paste relevant sections from manuscript title (include quotes in quotation marks "like this" to indicate direct quotes from your manuscript), or elaborate on this item by providing additional information not in the ms, or briefly explain why the item is not applicable/relevant for your study

Effect of a Mobile Application on Preoperative Patient Preparation for "Major Ambulatory Surgery": Protocol for a Randomized Clinical Trial

## 1b) ABSTRACT: Structured summary of trial design, methods, results, and conclusions

NPT extension: Description of experimental treatment, comparator, care providers, centers, and blinding status.

## 1b-i) Key features/functionalities/components of the intervention and comparator in the METHODS section of the ABSTRACT

Mention key features/functionalities/components of the intervention and comparator in the abstract. If possible, also mention theories and principles used for designing the site. Keep in mind the needs of systematic reviewers and indexers by including important synonyms. (Note: Only report in the abstract what the main paper is reporting. If this information is missing from the main body of text, consider adding it)

| | 1 | 2 | 3 | 4 | 5 | |
|---|---|---|---|---|---|---|
| subitem not at all important | ○ | ○ | ○ | ○ | ◉ | essential |

## Does your paper address subitem 1b-i? *

Copy and paste relevant sections from the manuscript abstract (include quotes in quotation marks "like this" to indicate direct quotes from your manuscript), or elaborate on this item by providing additional information not in the ms, or briefly explain why the item is not applicable/relevant for your study

We mention that Listeo+ is "a specific mobile Health App for MAS [Major Ambulatory Surgery] preoperative patient monitoring". We also included the outcomes: "The primary efficacy outcome is the level of compliance with preoperative recommendations. Secondary outcomes include the rate of cancellations, associated resource consumption, and perceived usability and utility with Listeo+ by participants of the intervention group"

## 1b-ii) Level of human involvement in the METHODS section of the ABSTRACT

Clarify the level of human involvement in the abstract, e.g., use phrases like “fully automated” vs. “therapist/nurse/care provider/physician-assisted” (mention number and expertise of providers involved, if any). (Note: Only report in the abstract what the main paper is reporting. If this information is missing from the main body of text, consider adding it)

| | 1 | 2 | 3 | 4 | 5 | |
|---|---|---|---|---|---|---|
| subitem not at all important | ○ | ○ | ○ | ◉ | ○ | essential |

## Does your paper address subitem 1b-ii?

Copy and paste relevant sections from the manuscript abstract (include quotes in quotation marks "like this" to indicate direct quotes from your manuscript), or elaborate on this item by providing additional information not in the ms, or briefly explain why the item is not applicable/relevant for your study

Listeo+ Mobile app has been designed for the "Preoperative Patient Preparation" (PPP) in Major Ambulatory Surgery. According to the literature, The PPP implies always patients, care-givers and health professionals and the Listeo+ targets the way to improve the interaction of all actors during that crucial procedure phase.

## 1b-iii) Open vs. closed, web-based (self-assessment) vs. face-to-face assessments in the METHODS section of the ABSTRACT

Mention how participants were recruited (online vs. offline), e.g., from an open access website or from a clinic or a closed online user group (closed usergroup trial), and clarify if this was a purely web-based trial, or there were face-to-face components (as part of the intervention or for assessment). Clearly say if outcomes were self-assessed through questionnaires (as common in web-based trials). Note: In traditional offline trials, an open trial (open-label trial) is a type of clinical trial in which both the researchers and participants know which treatment is being administered. To avoid confusion, use “blinded” or “unblinded” to indicated the level of blinding instead of “open”, as “open” in web-based trials usually refers to “open access” (i.e. participants can self-enrol). (Note: Only report in the abstract what the main paper is reporting. If this information is missing from the main body of text, consider adding it)

| | 1 | 2 | 3 | 4 | 5 | |
|---|---|---|---|---|---|---|
| subitem not at all important | ○ | ○ | ○ | ◉ | ○ | essential |

## Does your paper address subitem 1b-iii?

Copy and paste relevant sections from the manuscript abstract (include quotes in quotation marks "like this" to indicate direct quotes from your manuscript), or elaborate on this item by providing additional information not in the ms, or briefly explain why the item is not applicable/relevant for your study

"The study will include patients aged ≥18 years with surgical indication for MAS who meet the necessary technological and connectivity requirements" thus, the recruitment is closed and will take place in a face-to-face appoinment during the surgery consultation.

## 1b-iv) RESULTS section in abstract must contain use data

Report number of participants enrolled/assessed in each group, the use/uptake of the intervention (e.g., attrition/adherence metrics, use over time, number of logins etc.), in addition to primary/secondary outcomes. (Note: Only report in the abstract what the main paper is reporting. If this information is missing from the main body of text, consider adding it)

| | 1 | 2 | 3 | 4 | 5 | |
|---|---|---|---|---|---|---|
| subitem not at all important | ○ | ○ | ○ | ○ | ● | essential |

## Does your paper address subitem 1b-iv?

Copy and paste relevant sections from the manuscript abstract (include quotes in quotation marks "like this" to indicate direct quotes from your manuscript), or elaborate on this item by providing additional information not in the ms, or briefly explain why the item is not applicable/relevant for your study

The expected number of participants in the trial is mentioned: "790 patients" as a whole for both intervention and control groups

## 1b-v) CONCLUSIONS/DISCUSSION in abstract for negative trials

Conclusions/Discussions in abstract for negative trials: Discuss the primary outcome - if the trial is negative (primary outcome not changed), and the intervention was not used, discuss whether negative results are attributable to lack of uptake and discuss reasons. (Note: Only report in the abstract what the main paper is reporting. If this information is missing from the main body of text, consider adding it)

| | 1 | 2 | 3 | 4 | 5 | |
|---|---|---|---|---|---|---|
| subitem not at all important | ● | ○ | ○ | ○ | ○ | essential |

## Does your paper address subitem 1b-v?

Copy and paste relevant sections from the manuscript abstract (include quotes in quotation marks "like this" to indicate direct quotes from your manuscript), or elaborate on this item by providing additional information not in the ms, or briefly explain why the item is not applicable/relevant for your study

This is a study protocol so the conclusions are the expected outcomes: "The app allows health professionals to monitor in real-time patients' preparation and critical pre-operative recommendations fulfillment. A reduction of avoidable pre-operative cancellations due to a lack or a poor patient preparation is expected. Self-assessed Web-based questionnaires and focus group will provide important information about the Listeo+ app perceived usability and utility from patients and care personnel"

# INTRODUCTION

## 2a) In INTRODUCTION: Scientific background and explanation of rationale

### 2a-i) Problem and the type of system/solution

Describe the problem and the type of system/solution that is object of the study: intended as stand-alone intervention vs. incorporated in broader health care program? Intended for a particular patient population? Goals of the intervention, e.g., being more cost-effective to other interventions, replace or complement other solutions? (Note: Details about the intervention are provided in "Methods" under 5)

| | 1 | 2 | 3 | 4 | 5 | |
|---|---|---|---|---|---|---|
| subitem not at all important | ○ | ○ | ○ | ○ | ◉ | essential |

## Does your paper address subitem 2a-i? *

Copy and paste relevant sections from the manuscript (include quotes in quotation marks "like this" to indicate direct quotes from your manuscript), or elaborate on this item by providing additional information not in the ms, or briefly explain why the item is not applicable/relevant for your study

As we mentioned, "poor compliance with preoperative recommendations" is a problem that affects all kind of surgical interventions so the proposed solution (Listeo+ app) has the potential to be escalated to a whole healthcare system, reducing costs related to day-of-surgery cancellations (not only in MAS): "Although the rate of cancellations in MAS is approximately 4% [13-14]— lower than that reported in other countries where cancellations on the day of surgery oscillate between 5 and 40% [10,15]—a quarter of those cancellations (27%) are due to poor compliance with preoperative recommendations and are therefore avoidable"

## 2a-ii) Scientific background, rationale: What is known about the (type of) system

Scientific background, rationale: What is known about the (type of) system that is the object of the study (be sure to discuss the use of similar systems for other conditions/diagnoses, if appropiate), motivation for the study, i.e. what are the reasons for and what is the context for this specific study, from which stakeholder viewpoint is the study performed, potential impact of findings [2]. Briefly justify the choice of the comparator.

| | 1 | 2 | 3 | 4 | 5 | |
|---|---|---|---|---|---|---|
| subitem not at all important | ○ | ○ | ○ | ○ | ◉ | essential |

## Does your paper address subitem 2a-ii? *

Copy and paste relevant sections from the manuscript (include quotes in quotation marks "like this" to indicate direct quotes from your manuscript), or elaborate on this item by providing additional information not in the ms, or briefly explain why the item is not applicable/relevant for your study

We would highlight 5 sections:
1. We address what literature says about the role of adverse events, that require special control and monitoring, in surgical cancellations: "The implications of surgery cancellations can be analyzed from the perspectives of health management and patient safety, since their effects on health resource consumption can be considered adverse events (AEs) that require control and monitoring [5-7]"
2. We contextualized the importance of Major Ambulatory Surgeries within the Spanish National Health System: "In Spain, it represents 62.5% of the total number of surgeries performed by the National Health System (NHS) [11], which is one of the highest rates among Organisation for Economic Co-Operation and Development (OECD) member countries [12]"
3. Tools that are designed to "minimize risks in the preoperative process by assessing the patient's anesthetic risk [19] or verifying compliance with essential surgical aspects from the beginning to the end of surgery [20]" such as preassessment clinics and surgical safety checklists are also mentioned.
4. We stressed that there is "no evidence of the impact of good compliance by patients with preoperative recommendations" so the information provided by the sudy will significantly contribute to the state of the art.
5. We finnally focused on the Electronic Health Record and mHealth technologies intersection potential: "Some experiences based on information and communication technologies (ICT), such as incorporating the SSC in digital form to the Electronic Health Record (EHR) or sending text messages as a reminder of health appointments, have made it possible to improve compliance to treatment and surgical recommendations, reduce cancellations, and avoid no-shows [1,24-27]." [...] "Some mHealth-based interventions, such as the use of mobile Health Apps, have proven effective in the management of chronic diseases (e.g., diabetes, asthma, hypertension) by improving clinical parameters, compliance, and reducing disease costs [31]. Despite their great potential, the few initiatives undertaken thus far in the ambulatory surgical setting have been limited to postoperative patient monitoring [32-33]" as well as the referenced population the study suitability: "Currently, mobile devices (tablet, mobile phone, and/or wearable) have a very high degree of penetration in Spain [28] and Andalusia in particular, with 70.9% of the population (some 4.8 million inhabitants) connecting to the Internet through these devices [29]"
[...]
"The learning process and the improvement in systems integration and interoperability can be used for other initiatives within the framework of mobile Health Apps in Andalusia, a region with a favorable environment for the development of initiatives based on new technologies and, by extension, to the [illegible]

## 2b) In INTRODUCTION: Specific objectives or hypotheses

### Does your paper address CONSORT subitem 2b? *

Copy and paste relevant sections from the manuscript (include quotes in quotation marks "like this" to indicate direct quotes from your manuscript), or elaborate on this item by providing additional information not in the ms, or briefly explain why the item is not applicable/relevant for your study

Yes: "The aim of this study is to evaluate the impact of Listeo+ as a complement to SOC in patient compliance with preoperative recommendations, surgery cancellations, and associated resource consumption in the ambulatory surgical setting, in a clinical context of real-world evidence (RWE), and to evaluate the user experience with the application (perceived usability and utility)"

# METHODS

## 3a) Description of trial design (such as parallel, factorial) including allocation ratio

### Does your paper address CONSORT subitem 3a? *

Copy and paste relevant sections from the manuscript (include quotes in quotation marks "like this" to indicate direct quotes from your manuscript), or elaborate on this item by providing additional information not in the ms, or briefly explain why the item is not applicable/relevant for your study

Yes: "A multicenter, randomized, and open-label clinical trial was planned to evaluate the Listeo+ mobile Health App as a complement to SOC in patients undergoing MAS. This study protocol considers guidelines and recommendations of the Standard Protocol Items: Recommendations for Interventional Trials (SPIRIT) [34] and Consolidated Standards of Reporting Trials (CONSORT) statements [35]"

## 3b) Important changes to methods after trial commencement (such as eligibility criteria), with reasons

## Does your paper address CONSORT subitem 3b? *

Copy and paste relevant sections from the manuscript (include quotes in quotation marks "like this" to indicate direct quotes from your manuscript), or elaborate on this item by providing additional information not in the ms, or briefly explain why the item is not applicable/relevant for your study

We are still in a early pilot phase but we are definetely taking that into account.

## 3b-i) Bug fixes, Downtimes, Content Changes

Bug fixes, Downtimes, Content Changes: ehealth systems are often dynamic systems. A description of changes to methods therefore also includes important changes made on the intervention or comparator during the trial (e.g., major bug fixes or changes in the functionality or content) (5-iii) and other “unexpected events” that may have influenced study design such as staff changes, system failures/downtimes, etc. [2].

| | 1 | 2 | 3 | 4 | 5 | |
|---|---|---|---|---|---|---|
| subitem not at all important | ○ | ○ | ○ | ○ | ◉ | essential |

## Does your paper address subitem 3b-i?

Copy and paste relevant sections from the manuscript (include quotes in quotation marks "like this" to indicate direct quotes from your manuscript), or elaborate on this item by providing additional information not in the ms, or briefly explain why the item is not applicable/relevant for your study

The continuous improvement is a huge part of the Listeo+ app vision. Many versions have been tested within the Lab so far (we are now in the version 2.2.3.2 of the app) and some of the secondary outcomes will be useful for that: "The information about mobile application usability by the intervention group will be collected through a modified version of the self-assessed mobile-based Computer System Usability Questionnaire (CSUQ) [40]. [...] A discussion group will be organized to collect qualitative information about the utility perceived by health professionals. Both research tools will provide very insightful information that will eventually lead to a changes in the Listeo+ functionality or content."

## 4a) Eligibility criteria for participants

## Does your paper address CONSORT subitem 4a? *

Copy and paste relevant sections from the manuscript (include quotes in quotation marks "like this" to indicate direct quotes from your manuscript), or elaborate on this item by providing additional information not in the ms, or briefly explain why the item is not applicable/relevant for your study

Yes. We have explicitly clarified the Eligibility criteria for participants: "Eligibility Criteria
Characteristics and Selection Criteria of Patients Undergoing MAS
The patients participating in this study will be adults aged 18 years or older at the start of the study, who will undergo MAS in the specialties of traumatology, orthopedic surgery, ophthalmology, or general surgery"

## 4a-i) Computer / Internet literacy

Computer / Internet literacy is often an implicit "de facto" eligibility criterion - this should be explicitly clarified.

| | 1 | 2 | 3 | 4 | 5 | |
|---|---|---|---|---|---|---|
| subitem not at all important | ○ | ○ | ○ | ○ | ◉ | essential |

## Does your paper address subitem 4a-i?

Copy and paste relevant sections from the manuscript (include quotes in quotation marks "like this" to indicate direct quotes from your manuscript), or elaborate on this item by providing additional information not in the ms, or briefly explain why the item is not applicable/relevant for your study

Yes, it is part of the inclusion criteria and researchers are in charged of :
"Inclusion Criteria
To participate in the study, the following inclusion criteria should be met: 1) autonomous patients or dependents of one or more caregivers to perform their preoperative preparation, with the necessary technological and connectivity resources (ie.: to dispose a smartphone or tablet mobile device with an Android or iOS operating system with an Internet connection; and familiarity with mobile technologies)." Familiarity with the use mobile technologies is measured through closed-ended questions regarding the frequent of use and type of apps they have. Nonetheless, "Those patients who are autonomous to perform their preoperative preparation and lack the technological requirements but have a caregiver with the necessary technological and connectivity resources who can supervise their preoperative preparation may also be included in the study."

## 4a-ii) Open vs. closed, web-based vs. face-to-face assessments:

Open vs. closed, web-based vs. face-to-face assessments: Mention how participants were recruited (online vs. offline), e.g., from an open access website or from a clinic, and clarify if this was a purely web-based trial, or there were face-to-face components (as part of the intervention or for assessment), i.e., to what degree got the study team to know the participant. In online-only trials, clarify if participants were quasi-anonymous and whether having multiple identities was possible or whether technical or logistical measures (e.g., cookies, email confirmation, phone calls) were used to detect/prevent these.

| | 1 | 2 | 3 | 4 | 5 | |
|---|---|---|---|---|---|---|
| subitem not at all important | ○ | ○ | ○ | ○ | ◉ | essential |

## Does your paper address subitem 4a-ii? *

Copy and paste relevant sections from the manuscript (include quotes in quotation marks "like this" to indicate direct quotes from your manuscript), or elaborate on this item by providing additional information not in the ms, or briefly explain why the item is not applicable/relevant for your study

Yes. We have explicitly mentioned that patients are recruited in face-to-face appointment with doctors: "The patient flow from the MAS assessment appointment to hospital discharge is represented in the flowchart in Figure 1. This includes a first visit to surgery consultation; a second face-to-face anesthesia consultation (all patients except for ophthalmological patients with indication for topical anesthesia); and a third hospitalization visit to undergo MAS. Patients in the control group will follow the existing MAS patient assistance route in the centers, which consists in providing written recommendations. "

## 4a-iii) Information giving during recruitment

Information given during recruitment. Specify how participants were briefed for recruitment and in the informed consent procedures (e.g., publish the informed consent documentation as appendix, see also item X26), as this information may have an effect on user self-selection, user expectation and may also bias results.

| | 1 | 2 | 3 | 4 | 5 | |
|---|---|---|---|---|---|---|
| subitem not at all important | ○ | ○ | ○ | ○ | ◉ | essential |

## Does your paper address subitem 4a-iii?

Copy and paste relevant sections from the manuscript (include quotes in quotation marks "like this" to indicate direct quotes from your manuscript), or elaborate on this item by providing additional information not in the ms, or briefly explain why the item is not applicable/relevant for your study

Yes. The trial objectives will be explained by researchers in the face-to-face surgical consultation and a very clear informed consent will be provided:
"Allocation
Subjects who meet the inclusion criteria and sign informed consent will be provided with an information sheet about the project and evaluated before participating in the study"

## 4b) Settings and locations where the data were collected

## Does your paper address CONSORT subitem 4b? *

Copy and paste relevant sections from the manuscript (include quotes in quotation marks "like this" to indicate direct quotes from your manuscript), or elaborate on this item by providing additional information not in the ms, or briefly explain why the item is not applicable/relevant for your study

Yes. "Study Setting
Four High Resolution Hospital Centers (HARCs) belonging to the Public Health System of Andalusia as a part of the NHS hospital network are recruiting patients for this study. HARCs encourage ambulatory surgery and short-term hospitalization using MAS, so they were considered suitable centers to evaluate the initiative. Participating Hospital reference population is about 187,957 inhabitants."

## 4b-i) Report if outcomes were (self-)assessed through online questionnaires

Clearly report if outcomes were (self-)assessed through online questionnaires (as common in web-based trials) or otherwise.

| | 1 | 2 | 3 | 4 | 5 | |
|---|---|---|---|---|---|---|
| subitem not at all important | ○ | ○ | ○ | ○ | ◉ | essential |

## Does your paper address subitem 4b-i? *

Copy and paste relevant sections from the manuscript (include quotes in quotation marks "like this" to indicate direct quotes from your manuscript), or elaborate on this item by providing additional information not in the ms, or briefly explain why the item is not applicable/relevant for your study

Yes: "The information about mobile application usability by the intervention group will be collected through a modified version of the self-assessed mobile-based Computer System Usability Questionnaire (CSUQ) [40]. "

## 4b-ii) Report how institutional affiliations are displayed

Report how institutional affiliations are displayed to potential participants [on ehealth media], as affiliations with prestigious hospitals or universities may affect volunteer rates, use, and reactions with regards to an intervention.(Not a required item – describe only if this may bias results)

| | 1 | 2 | 3 | 4 | 5 | |
|---|---|---|---|---|---|---|
| subitem not at all important | ○ | ○ | ● | ○ | ○ | essential |

## Does your paper address subitem 4b-ii?

Copy and paste relevant sections from the manuscript (include quotes in quotation marks "like this" to indicate direct quotes from your manuscript), or elaborate on this item by providing additional information not in the ms, or briefly explain why the item is not applicable/relevant for your study

Not in the paper but it's included in the Information Consent Procedure that is provided to the participants. It explains that this is a trial conducted by their healthcare provider, belonging to the Healthcare Regional Ministry.

## 5) The interventions for each group with sufficient details to allow replication, including how and when they were actually administered

## 5-i) Mention names, credential, affiliations of the developers, sponsors, and owners

Mention names, credential, affiliations of the developers, sponsors, and owners [6] (if authors/evaluators are owners or developer of the software, this needs to be declared in a “Conflict of interest” section or mentioned elsewhere in the manuscript).

| | 1 | 2 | 3 | 4 | 5 | |
|---|---|---|---|---|---|---|
| subitem not at all important | ○ | ○ | ○ | ○ | ● | essential |

## Does your paper address subitem 5-i?

Copy and paste relevant sections from the manuscript (include quotes in quotation marks "like this" to indicate direct quotes from your manuscript), or elaborate on this item by providing additional information not in the ms, or briefly explain why the item is not applicable/relevant for your study

Yes "Conflicts of Interest
The authors actively participated in the design of the intervention (Listeo+ mobile health app) but have no conflict of interest affecting finances due to the app copyright belongs to the Andalusian Regional Healthcare Ministry."

## 5-ii) Describe the history/development process

Describe the history/development process of the application and previous formative evaluations (e.g., focus groups, usability testing), as these will have an impact on adoption/use rates and help with interpreting results.

| | 1 | 2 | 3 | 4 | 5 | |
|---|---|---|---|---|---|---|
| subitem not at all important | ○ | ○ | ● | ○ | ○ | essential |

## Does your paper address subitem 5-ii?

Copy and paste relevant sections from the manuscript (include quotes in quotation marks "like this" to indicate direct quotes from your manuscript), or elaborate on this item by providing additional information not in the ms, or briefly explain why the item is not applicable/relevant for your study

This is a study protocol that includes that phase.

## 5-iii) Revisions and updating

Revisions and updating. Clearly mention the date and/or version number of the application/intervention (and comparator, if applicable) evaluated, or describe whether the intervention underwent major changes during the evaluation process, or whether the development and/or content was “frozen” during the trial. Describe dynamic components such as news feeds or changing content which may have an impact on the replicability of the intervention (for unexpected events see item 3b).

| | 1 | 2 | 3 | 4 | 5 | |
|---|---|---|---|---|---|---|
| subitem not at all important | ○ | ○ | ○ | ● | ○ | essential |

## Does your paper address subitem 5-iii?

Copy and paste relevant sections from the manuscript (include quotes in quotation marks "like this" to indicate direct quotes from your manuscript), or elaborate on this item by providing additional information not in the ms, or briefly explain why the item is not applicable/relevant for your study

Yes. The version of the Listeo+ mobile app that is being tested is mentioned: "v.2.2.3.2"

## 5-iv) Quality assurance methods

Provide information on quality assurance methods to ensure accuracy and quality of information provided [1], if applicable.

| | 1 | 2 | 3 | 4 | 5 | |
|---|---|---|---|---|---|---|
| subitem not at all important | ○ | ○ | ○ | ○ | ◉ | essential |

## Does your paper address subitem 5-iv?

Copy and paste relevant sections from the manuscript (include quotes in quotation marks "like this" to indicate direct quotes from your manuscript), or elaborate on this item by providing additional information not in the ms, or briefly explain why the item is not applicable/relevant for your study

Yes. Specific tools were took into account for that:
"Data Monitoring and Validation
An electronic monitoring of the completion of the eCRFs will be performed to detect missing information and possible data inconsistencies, thus ensuring their quality. For this purpose, the researchers will be contacted during the patient recruitment phase, three and six months after the start of the project, using confidential information access codes. The inclusion of patients according to the established criteria (inclusion/exclusion criteria), the correct completion of the eCRFs, the signing and filing of the informed consent form of the participating patients, and any other aspects required by the research team will be reviewed. The monitor will communicate to the corresponding research team the variables that must be reviewed in cases of lost or inconsistent data."

## 5-v) Ensure replicability by publishing the source code, and/or providing screenshots/screen-capture video, and/or providing flowcharts of the algorithms used

Ensure replicability by publishing the source code, and/or providing screenshots/screen-capture video, and/or providing flowcharts of the algorithms used. Replicability (i.e., other researchers should in principle be able to replicate the study) is a hallmark of scientific reporting.

| | 1 | 2 | 3 | 4 | 5 | |
|---|---|---|---|---|---|---|
| subitem not at all important | ○ | ○ | ○ | ◉ | ○ | essential |

## Does your paper address subitem 5-v?

Copy and paste relevant sections from the manuscript (include quotes in quotation marks "like this" to indicate direct quotes from your manuscript), or elaborate on this item by providing additional information not in the ms, or briefly explain why the item is not applicable/relevant for your study

Yes. We provided screenshots and flowcharts. Once the efficacy is going to be proven we want to openly publish the source code

## 5-vi) Digital preservation

Digital preservation: Provide the URL of the application, but as the intervention is likely to change or disappear over the course of the years; also make sure the intervention is archived (Internet Archive, webcitation.org, and/or publishing the source code or screenshots/videos alongside the article). As pages behind login screens cannot be archived, consider creating demo pages which are accessible without login.

| | 1 | 2 | 3 | 4 | 5 | |
|---|---|---|---|---|---|---|
| subitem not at all important | ○ | ○ | ◉ | ○ | ○ | essential |

## Does your paper address subitem 5-vi?

Copy and paste relevant sections from the manuscript (include quotes in quotation marks "like this" to indicate direct quotes from your manuscript), or elaborate on this item by providing additional information not in the ms, or briefly explain why the item is not applicable/relevant for your study

We are working hard to archive and store all the information related to the project as well as a stable URL.

## 5-vii) Access

Access: Describe how participants accessed the application, in what setting/context, if they had to pay (or were paid) or not, whether they had to be a member of specific group. If known, describe how participants obtained “access to the platform and Internet” [1]. To ensure access for editors/reviewers/readers, consider to provide a “backdoor” login account or demo mode for reviewers/readers to explore the application (also important for archiving purposes, see vi).

| | 1 | 2 | 3 | 4 | 5 | |
|---|---|---|---|---|---|---|
| subitem not at all important | ○ | ○ | ○ | ○ | ◉ | essential |

## Does your paper address subitem 5-vii? *

Copy and paste relevant sections from the manuscript (include quotes in quotation marks "like this" to indicate direct quotes from your manuscript), or elaborate on this item by providing additional information not in the ms, or briefly explain why the item is not applicable/relevant for your study

Yes, the access to the application by participants is explained: "Participants are not going to pay for the app."
"Patients included in the intervention group will be provided with personal recommendations through the Listeo+ mobile app (Figure 2 and Figure 3). They will be given access to download the mobile application through their mobile apps market (Google Play) using a link and/or QR code.
Once they download the app, patients will be able to use a personalized QR that will allows them to access the episode identification data and specific recommendations for that particular case that is already set up in the web-based Listeo+ module integrated in the EHR Hospitals platform."

## 5-viii) Mode of delivery, features/functionalities/components of the intervention and comparator, and the theoretical framework

Describe mode of delivery, features/functionalities/components of the intervention and comparator, and the theoretical framework [6] used to design them (instructional strategy [1], behaviour change techniques, persuasive features, etc., see e.g., [7, 8] for terminology). This includes an in-depth description of the content (including where it is coming from and who developed it) [1]," whether [and how] it is tailored to individual circumstances and allows users to track their progress and receive feedback" [6]. This also includes a description of communication delivery channels and – if computer-mediated communication is a component – whether communication was synchronous or asynchronous [6]. It also includes information on presentation strategies [1], including page design principles, average amount of text on pages, presence of hyperlinks to other resources, etc. [1].

| | 1 | 2 | 3 | 4 | 5 | |
|---|---|---|---|---|---|---|
| subitem not at all important | ○ | ○ | ○ | ○ | ◉ | essential |

## Does your paper address subitem 5-viii? *

Copy and paste relevant sections from the manuscript (include quotes in quotation marks "like this" to indicate direct quotes from your manuscript), or elaborate on this item by providing additional information not in the ms, or briefly explain why the item is not applicable/relevant for your study

The Listeo+ app is designed to keep a synchronic communication between patients and health personnel.

## 5-ix) Describe use parameters

Describe use parameters (e.g., intended “doses” and optimal timing for use). Clarify what instructions or recommendations were given to the user, e.g., regarding timing, frequency, heaviness of use, if any, or was the intervention used ad libitum.

| | 1 | 2 | 3 | 4 | 5 | |
|---|---|---|---|---|---|---|
| subitem not at all important | ○ | ○ | ○ | ◉ | ○ | essential |

## Does your paper address subitem 5-ix?

Copy and paste relevant sections from the manuscript (include quotes in quotation marks "like this" to indicate direct quotes from your manuscript), or elaborate on this item by providing additional information not in the ms, or briefly explain why the item is not applicable/relevant for your study

Yes. Instructions will be given to the participants: "Any interaction of the app users with the individualized recommendations will be notify to the researchers servers and visualize in the EHR platform, which will automatically send notifications to the mobile application as well as emails to the designated healthcare professional of the participating center in case a critical recommendation (recommendations which may pose a risk to the patient or in which patient non-compliance may lead to the suspension, cancellation, or rescheduling of surgery as stated in table 1) is not marked."

## 5-x) Clarify the level of human involvement

Clarify the level of human involvement (care providers or health professionals, also technical assistance) in the e-intervention or as co-intervention (detail number and expertise of professionals involved, if any, as well as “type of assistance offered, the timing and frequency of the support, how it is initiated, and the medium by which the assistance is delivered”. It may be necessary to distinguish between the level of human involvement required for the trial, and the level of human involvement required for a routine application outside of a RCT setting (discuss under item 21 – generalizability).

| | 1 | 2 | 3 | 4 | 5 | |
|---|---|---|---|---|---|---|
| subitem not at all important | ○ | ○ | ○ | ◉ | ○ | essential |

## Does your paper address subitem 5-x?

Copy and paste relevant sections from the manuscript (include quotes in quotation marks "like this" to indicate direct quotes from your manuscript), or elaborate on this item by providing additional information not in the ms, or briefly explain why the item is not applicable/relevant for your study

Yes. The human involvement is explained: "Any interaction of the app users with the individualized recommendations will be notify to the researchers servers and visualize in the EHR platform, which will automatically send notifications to the mobile application as well as emails to the designated healthcare professional of the participating center in case a critical recommendation (recommendations which may pose a risk to the patient or in which patient non-compliance may lead to the suspension, cancellation, or rescheduling of surgery as stated in table 1) is not marked. Additionally, healthcare professionals will be able to contact by phone to encourage patients to meet the recommendation or solve any potential problem. The nature of the incident will be always registered. "

## 5-xi) Report any prompts/reminders used

Report any prompts/reminders used: Clarify if there were prompts (letters, emails, phone calls, SMS) to use the application, what triggered them, frequency etc. It may be necessary to distinguish between the level of prompts/reminders required for the trial, and the level of prompts/reminders for a routine application outside of a RCT setting (discuss under item 21 – generalizability).

| | 1 | 2 | 3 | 4 | 5 | |
|---|---|---|---|---|---|---|
| subitem not at all important | ○ | ○ | ○ | ○ | ◉ | essential |

## Does your paper address subitem 5-xi? *

Copy and paste relevant sections from the manuscript (include quotes in quotation marks "like this" to indicate direct quotes from your manuscript), or elaborate on this item by providing additional information not in the ms, or briefly explain why the item is not applicable/relevant for your study

Yes. As mentioned before, this is a crucial part of the intervention: "Any interaction of the app users with the individualized recommendations will be notify to the researchers servers and visualize in the EHR platform, which will automatically send notifications to the mobile application as well as emails to the designated healthcare professional of the participating center in case a critical recommendation (recommendations which may pose a risk to the patient or in which patient non-compliance may lead to the suspension, cancellation, or rescheduling of surgery as stated in table 1) is not marked. Additionally, healthcare professionals will be able to contact by phone to encourage patients to meet the recommendation or solve any potential problem. The nature of the incident will be always registered. "

## 5-xii) Describe any co-interventions (incl. training/support)

Describe any co-interventions (incl. training/support): Clearly state any interventions that are provided in addition to the targeted eHealth intervention, as ehealth intervention may not be designed as stand-alone intervention. This includes training sessions and support [1]. It may be necessary to distinguish between the level of training required for the trial, and the level of training for a routine application outside of a RCT setting (discuss under item 21 – generalizability.

| | 1 | 2 | 3 | 4 | 5 | |
|---|---|---|---|---|---|---|
| subitem not at all important | ○ | ○ | ◉ | ○ | ○ | essential |

## Does your paper address subitem 5-xii? *

Copy and paste relevant sections from the manuscript (include quotes in quotation marks "like this" to indicate direct quotes from your manuscript), or elaborate on this item by providing additional information not in the ms, or briefly explain why the item is not applicable/relevant for your study

Yes. Since this trial is designed to be fully implemented in the surgical units everyday life, no specific patients training is going to be conducted. Participants receive the instructions verbally and with the informed consent. "Subjects who meet the inclusion criteria and sign informed consent will be provided with an information sheet about the project and evaluated before participating in the study".

## 6a) Completely defined pre-specified primary and secondary outcome measures, including how and when they were assessed

## Does your paper address CONSORT subitem 6a? *

Copy and paste relevant sections from the manuscript (include quotes in quotation marks "like this" to indicate direct quotes from your manuscript), or elaborate on this item by providing additional information not in the ms, or briefly explain why the item is not applicable/relevant for your study

Yes

## 6a-i) Online questionnaires: describe if they were validated for online use and apply CHERRIES items to describe how the questionnaires were designed/deployed

If outcomes were obtained through online questionnaires, describe if they were validated for online use and apply CHERRIES items to describe how the questionnaires were designed/deployed [9].

| | 1 | 2 | 3 | 4 | 5 | |
|---|---|---|---|---|---|---|
| subitem not at all important | ○ | ◉ | ○ | ○ | ○ | essential |

## Does your paper address subitem 6a-i?

Copy and paste relevant sections from manuscript text

The Computer System Usability Questionnaire (CSUQ) is specifically designed to be self-administered. Thus, CHERRIES checklist was not needed.

## 6a-ii) Describe whether and how “use” (including intensity of use/dosage) was defined/measured/monitored

Describe whether and how “use” (including intensity of use/dosage) was defined/measured/monitored (logins, logfile analysis, etc.). Use/adoption metrics are important process outcomes that should be reported in any ehealth trial.

| | 1 | 2 | 3 | 4 | 5 | |
|---|---|---|---|---|---|---|
| subitem not at all important | ○ | ◉ | ○ | ○ | ○ | essential |

## Does your paper address subitem 6a-ii?

Copy and paste relevant sections from manuscript text

Logins and Logfile analysis metrics are not priorities since the primary outcomes is preoperative recomendations compliance.

## 6a-iii) Describe whether, how, and when qualitative feedback from participants was obtained

Describe whether, how, and when qualitative feedback from participants was obtained (e.g., through emails, feedback forms, interviews, focus groups).

| | 1 | 2 | 3 | 4 | 5 | |
|---|---|---|---|---|---|---|
| subitem not at all important | ○ | ○ | ○ | ○ | ◉ | essential |

## Does your paper address subitem 6a-iii?

Copy and paste relevant sections from manuscript text

Yes. "Four focus groups –6 to 8 participants each-, will be organized to collect qualitative data about the utility perceived by health professionals as well as intervention group patients experience with the app.
Both research tools will provide very insightful information that will eventually lead to a changes in the Listeo+ functionality or content."

## 6b) Any changes to trial outcomes after the trial commenced, with reasons

### Does your paper address CONSORT subitem 6b? *

Copy and paste relevant sections from the manuscript (include quotes in quotation marks "like this" to indicate direct quotes from your manuscript), or elaborate on this item by providing additional information not in the ms, or briefly explain why the item is not applicable/relevant for your study

This is a study protocol. Not required

## 7a) How sample size was determined

NPT: When applicable, details of whether and how the clustering by care provides or centers was addressed

### 7a-i) Describe whether and how expected attrition was taken into account when calculating the sample size

Describe whether and how expected attrition was taken into account when calculating the sample size.

| | 1 | 2 | 3 | 4 | 5 | |
|---|---|---|---|---|---|---|
| subitem not at all important | ○ | ○ | ○ | ○ | ◉ | essential |

### Does your paper address subitem 7a-i?

Copy and paste relevant sections from manuscript title (include quotes in quotation marks "like this" to indicate direct quotes from your manuscript), or elaborate on this item by providing additional information not in the ms, or briefly explain why the item is not applicable/relevant for your study

Yes. "The sample size required to estimate the main objective of the study was calculated as 395 patients per group for a total of 790 patients. This estimate was made assuming an alpha risk of 0.05, a beta risk of 0.2, and a difference in proportions between the groups of 10%. The analysis will be performed considering per intention to treat (ITT) and per protocol (PP) populations. The ITT population will include all randomized patients, whereas the PP population will include randomized patients who finally obtain an appointment to undergo MAS."

## 7b) When applicable, explanation of any interim analyses and stopping guidelines

## Does your paper address CONSORT subitem 7b? *

Copy and paste relevant sections from the manuscript (include quotes in quotation marks "like this" to indicate direct quotes from your manuscript), or elaborate on this item by providing additional information not in the ms, or briefly explain why the item is not applicable/relevant for your study

Not applicable.

## 8a) Method used to generate the random allocation sequence

NPT: When applicable, how care providers were allocated to each trial group

## Does your paper address CONSORT subitem 8a? *

Copy and paste relevant sections from the manuscript (include quotes in quotation marks "like this" to indicate direct quotes from your manuscript), or elaborate on this item by providing additional information not in the ms, or briefly explain why the item is not applicable/relevant for your study

Yes. "To allocate the patients to study groups, simple randomization 1:1 procedure will be used. To include the patients in the study, each center will be provided one randomization scheme generated by computer. Given the characteristics of the study, it is not possible to blind the patients and professionals. Subsequently, we will collect sociodemographic data (age, sex, area of residence of the patient, level of studies of the patient/caregiver using the application or patient/caregiver of the control group, occupation, marital status, knowledge/handling of apps); clinical data (type of surgery, medical diagnosis (ICD-9), anesthetic evaluation, medications taken); and functional situation by measuring disability (Barthel Index)."

## 8b) Type of randomisation; details of any restriction (such as blocking and block size)

## Does your paper address CONSORT subitem 8b? *

Copy and paste relevant sections from the manuscript (include quotes in quotation marks "like this" to indicate direct quotes from your manuscript), or elaborate on this item by providing additional information not in the ms, or briefly explain why the item is not applicable/relevant for your study

Yes. "The sample size required to estimate the main objective of the study was calculated as 395 patients per group for a total of 790 patients."

## 9) Mechanism used to implement the random allocation sequence (such as sequentially numbered containers), describing any steps taken to conceal the sequence until interventions were assigned

### Does your paper address CONSORT subitem 9? *

Copy and paste relevant sections from the manuscript (include quotes in quotation marks "like this" to indicate direct quotes from your manuscript), or elaborate on this item by providing additional information not in the ms, or briefly explain why the item is not applicable/relevant for your study

Yes. "To allocate the patients to study groups, simple randomization 1:1 procedure will be used. To include the patients in the study, each center will be provided one randomization scheme generated by computer."

## 10) Who generated the random allocation sequence, who enrolled participants, and who assigned participants to interventions

### Does your paper address CONSORT subitem 10? *

Copy and paste relevant sections from the manuscript (include quotes in quotation marks "like this" to indicate direct quotes from your manuscript), or elaborate on this item by providing additional information not in the ms, or briefly explain why the item is not applicable/relevant for your study

Yes. "To include the patients in the study, each center will be provided one randomization scheme generated by computer.""

## 11a) If done, who was blinded after assignment to interventions (for example, participants, care providers, those assessing outcomes) and how

NPT: Whether or not administering co-interventions were blinded to group assignment

### 11a-i) Specify who was blinded, and who wasn’t

Specify who was blinded, and who wasn’t. Usually, in web-based trials it is not possible to blind the participants [1, 3] (this should be clearly acknowledged), but it may be possible to blind outcome assessors, those doing data analysis or those administering co-interventions (if any).

| | 1 | 2 | 3 | 4 | 5 | |
|---|---|---|---|---|---|---|
| subitem not at all important | ○ | ○ | ● | ○ | ○ | essential |

## Does your paper address subitem 11a-i? *

Copy and paste relevant sections from the manuscript (include quotes in quotation marks "like this" to indicate direct quotes from your manuscript), or elaborate on this item by providing additional information not in the ms, or briefly explain why the item is not applicable/relevant for your study

Blinding was not possible in this study

## 11a-ii) Discuss e.g., whether participants knew which intervention was the "intervention of interest" and which one was the "comparator"

Informed consent procedures (4a-ii) can create biases and certain expectations - discuss e.g., whether participants knew which intervention was the "intervention of interest" and which one was the "comparator".

| | 1 | 2 | 3 | 4 | 5 | |
|---|---|---|---|---|---|---|
| subitem not at all important | ○ | ○ | ○ | ○ | ◉ | essential |

## Does your paper address subitem 11a-ii?

Copy and paste relevant sections from the manuscript (include quotes in quotation marks "like this" to indicate direct quotes from your manuscript), or elaborate on this item by providing additional information not in the ms, or briefly explain why the item is not applicable/relevant for your study

Yes. It is specified in the informed consent.

## 11b) If relevant, description of the similarity of interventions

(this item is usually not relevant for ehealth trials as it refers to similarity of a placebo or sham intervention to a active medication/intervention)

## Does your paper address CONSORT subitem 11b? *

Copy and paste relevant sections from the manuscript (include quotes in quotation marks "like this" to indicate direct quotes from your manuscript), or elaborate on this item by providing additional information not in the ms, or briefly explain why the item is not applicable/relevant for your study

Not applicable.

## 12a) Statistical methods used to compare groups for primary and secondary outcomes

NPT: When applicable, details of whether and how the clustering by care providers or centers was addressed

## Does your paper address CONSORT subitem 12a? *

Copy and paste relevant sections from the manuscript (include quotes in quotation marks "like this" to indicate direct quotes from your manuscript), or elaborate on this item by providing additional information not in the ms, or briefly explain why the item is not applicable/relevant for your study

Yes. Statistical analyses will be performed using the R software version 3.3.2. For all analyses, an alpha (α) risk of 0.05 will be assumed; therefore, to consider a statistically significant difference, the p value of the contrast statistic should be ≤0.05. The statistical analysis planned a priori will consist of a descriptive analysis of the demographic and clinical characteristics of the patients. For the quantitative variables, the mean, standard deviation, 95% confidence intervals (95% CI), variance, standard error, 5% trimmed mean, median, minimum, and maximum will be calculated. For the qualitative variables, frequency distributions with their respective percentages will be calculated. To determine whether there are differences in the level of compliance with surgical recommendations between the group with written recommendations and the group with written recommendations plus the app, Fisher's exact test will be carried out."

## 12a-i) Imputation techniques to deal with attrition / missing values

Imputation techniques to deal with attrition / missing values: Not all participants will use the intervention/comparator as intended and attrition is typically high in ehealth trials. Specify how participants who did not use the application or dropped out from the trial were treated in the statistical analysis (a complete case analysis is strongly discouraged, and simple imputation techniques such as LOCF may also be problematic [4]).

| | 1 | 2 | 3 | 4 | 5 | |
|---|---|---|---|---|---|---|
| subitem not at all important | ○ | ● | ○ | ○ | ○ | essential |

## Does your paper address subitem 12a-i? *

Copy and paste relevant sections from the manuscript (include quotes in quotation marks "like this" to indicate direct quotes from your manuscript), or elaborate on this item by providing additional information not in the ms, or briefly explain why the item is not applicable/relevant for your study

In our study, the source of missing values that could affect the objective assement is the surgery cancellations. But in our case any outcome of the surgery has assigned a value (cancelled or not).

## 12b) Methods for additional analyses, such as subgroup analyses and adjusted analyses

## Does your paper address CONSORT subitem 12b? *

Copy and paste relevant sections from the manuscript (include quotes in quotation marks "like this" to indicate direct quotes from your manuscript), or elaborate on this item by providing additional information not in the ms, or briefly explain why the item is not applicable/relevant for your study

Yes. Subgroups analyses with the care givers group has been taken into account.

## X26) REB/IRB Approval and Ethical Considerations [recommended as subheading under "Methods"] (not a CONSORT item)

## X26-i) Comment on ethics committee approval

| | 1 | 2 | 3 | 4 | 5 | |
|---|---|---|---|---|---|---|
| subitem not at all important | ○ | ○ | ○ | ○ | ◉ | essential |

## Does your paper address subitem X26-i?

Copy and paste relevant sections from the manuscript (include quotes in quotation marks "like this" to indicate direct quotes from your manuscript), or elaborate on this item by providing additional information not in the ms, or briefly explain why the item is not applicable/relevant for your study

Yes. "The study protocol has been evaluated and approved by the Regional Ethics Committee of Andalusia through the Biomedical Research Ethics Portal of Andalucía (PEIBA, for its acronym in Spanish). The present study will be conducted in accordance with the principles of the latest version of the Declaration of Helsinki and will follow the Good Clinical Practice guidelines of Spain."

## x26-ii) Outline informed consent procedures

Outline informed consent procedures e.g., if consent was obtained offline or online (how? Checkbox, etc.?), and what information was provided (see 4a-ii). See [6] for some items to be included in informed consent documents.

| | 1 | 2 | 3 | 4 | 5 | |
|---|---|---|---|---|---|---|
| subitem not at all important | ○ | ○ | ○ | ○ | ◉ | essential |

## Does your paper address subitem X26-ii?

Copy and paste relevant sections from the manuscript (include quotes in quotation marks "like this" to indicate direct quotes from your manuscript), or elaborate on this item by providing additional information not in the ms, or briefly explain why the item is not applicable/relevant for your study

The informed consent is obtained offline: "Subjects who meet the inclusion criteria and sign informed consent will be provided with an information sheet about the project and evaluated before participating in the study."

## X26-iii) Safety and security procedures

Safety and security procedures, incl. privacy considerations, and any steps taken to reduce the likelihood or detection of harm (e.g., education and training, availability of a hotline)

| | 1 | 2 | 3 | 4 | 5 | |
|---|---|---|---|---|---|---|
| subitem not at all important | ○ | ○ | ○ | ○ | ◉ | essential |

## Does your paper address subitem X26-iii?

Copy and paste relevant sections from the manuscript (include quotes in quotation marks "like this" to indicate direct quotes from your manuscript), or elaborate on this item by providing additional information not in the ms, or briefly explain why the item is not applicable/relevant for your study

Yes. A hotline telephone number is provided in the informed consent.

# RESULTS

## 13a) For each group, the numbers of participants who were randomly assigned, received intended treatment, and were analysed for the primary outcome

NPT: The number of care providers or centers performing the intervention in each group and the number of patients treated by each care provider in each center

## Does your paper address CONSORT subitem 13a? *

Copy and paste relevant sections from the manuscript (include quotes in quotation marks "like this" to indicate direct quotes from your manuscript), or elaborate on this item by providing additional information not in the ms, or briefly explain why the item is not applicable/relevant for your study

Yes. We specified the number of centers involved (4) and the number of expected participants (790) but we don't know yet how many patients are going to be recruited in each center

## 13b) For each group, losses and exclusions after randomisation, together with reasons

### Does your paper address CONSORT subitem 13b? (NOTE: Preferably, this is shown in a CONSORT flow diagram) *

Copy and paste relevant sections from the manuscript (include quotes in quotation marks "like this" to indicate direct quotes from your manuscript), or elaborate on this item by providing additional information not in the ms, or briefly explain why the item is not applicable/relevant for your study

Partially applicable. "To avoid loss of patients, a rescue procedure will be used with those patients who, within a period of 7 to 14 days, have not registered on the Listeo+ app. A telephone call will be made urging them to register on the Listeo+ app. If the patient has not registered within 72 hours (three days) after the rescue call has been made, the patient will be excluded from the study."

### 13b-i) Attrition diagram

Strongly recommended: An attrition diagram (e.g., proportion of participants still logging in or using the intervention/comparator in each group plotted over time, similar to a survival curve) or other figures or tables demonstrating usage/dose/engagement.

| | 1 | 2 | 3 | 4 | 5 | |
|---|---|---|---|---|---|---|
| subitem not at all important | ○ | ○ | ◉ | ○ | ○ | essential |

### Does your paper address subitem 13b-i?

Copy and paste relevant sections from the manuscript or cite the figure number if applicable (include quotes in quotation marks "like this" to indicate direct quotes from your manuscript), or elaborate on this item by providing additional information not in the ms, or briefly explain why the item is not applicable/relevant for your study

Yes. It's included in the "figure 1". Nonetheless, the proportion of participants still logging is not possible since this is still a study protocol

## 14a) Dates defining the periods of recruitment and follow-up

## Does your paper address CONSORT subitem 14a? *

Copy and paste relevant sections from the manuscript (include quotes in quotation marks "like this" to indicate direct quotes from your manuscript), or elaborate on this item by providing additional information not in the ms, or briefly explain why the item is not applicable/relevant for your study

Yes. "The data collection and cleaning phases are estimated to be completed in July 2018, and the analysis with the final results will be conducted in September 2018."

## 14a-i) Indicate if critical “secular events” fell into the study period

Indicate if critical “secular events” fell into the study period, e.g., significant changes in Internet resources available or “changes in computer hardware or Internet delivery resources”

| | 1 | 2 | 3 | 4 | 5 | |
|---|---|---|---|---|---|---|
| subitem not at all important | (●) | ( ) | ( ) | ( ) | ( ) | essential |

## Does your paper address subitem 14a-i?

Copy and paste relevant sections from the manuscript (include quotes in quotation marks "like this" to indicate direct quotes from your manuscript), or elaborate on this item by providing additional information not in the ms, or briefly explain why the item is not applicable/relevant for your study

Not applicable

## 14b) Why the trial ended or was stopped (early)

## Does your paper address CONSORT subitem 14b? *

Copy and paste relevant sections from the manuscript (include quotes in quotation marks "like this" to indicate direct quotes from your manuscript), or elaborate on this item by providing additional information not in the ms, or briefly explain why the item is not applicable/relevant for your study

Not applicable

## 15) A table showing baseline demographic and clinical characteristics for each group

NPT: When applicable, a description of care providers (case volume, qualification, expertise, etc.) and centers (volume) in each group

## Does your paper address CONSORT subitem 15? *

Copy and paste relevant sections from the manuscript (include quotes in quotation marks "like this" to indicate direct quotes from your manuscript), or elaborate on this item by providing additional information not in the ms, or briefly explain why the item is not applicable/relevant for your study

Yes. The study considers control variables: "To assess the influence of sociodemographic and clinical characteristics of the patients in the level of compliance with preoperative recommendations a multivariate logistic regression will be performed. The reasons for exclusion of the ITT population will be included."

## 15-i) Report demographics associated with digital divide issues

In ehealth trials it is particularly important to report demographics associated with digital divide issues, such as age, education, gender, social-economic status, computer/Internet/ehealth literacy of the participants, if known.

| | 1 | 2 | 3 | 4 | 5 | |
|---|---|---|---|---|---|---|
| subitem not at all important | ○ | ○ | ○ | ○ | ◉ | essential |

## Does your paper address subitem 15-i? *

Copy and paste relevant sections from the manuscript (include quotes in quotation marks "like this" to indicate direct quotes from your manuscript), or elaborate on this item by providing additional information not in the ms, or briefly explain why the item is not applicable/relevant for your study

Yes. "Given the characteristics of the study, it is not possible to blind the patients and professionals. Subsequently, we will collect sociodemographic data (age, sex, area of residence of the patient, level of education of the patient/caregiver using the application or patient/caregiver of the control group, occupation, marital status, knowledge/handling of apps); clinical data (type of surgery, medical diagnosis (ICD-9), anesthetic evaluation, medications taken); and functional situation by measuring disability (Barthel Index)."

## 16) For each group, number of participants (denominator) included in each analysis and whether the analysis was by original assigned groups

## 16-i) Report multiple “denominators” and provide definitions

Report multiple “denominators” and provide definitions: Report N’s (and effect sizes) “across a range of study participation [and use] thresholds” [1], e.g., N exposed, N consented, N used more than x times, N used more than y weeks, N participants “used” the intervention/comparator at specific pre-defined time points of interest (in absolute and relative numbers per group). Always clearly define “use” of the intervention.

| | 1 | 2 | 3 | 4 | 5 | |
|---|---|---|---|---|---|---|
| subitem not at all important | ○ | ○ | ◉ | ○ | ○ | essential |

## Does your paper address subitem 16-i? *

Copy and paste relevant sections from the manuscript (include quotes in quotation marks "like this" to indicate direct quotes from your manuscript), or elaborate on this item by providing additional information not in the ms, or briefly explain why the item is not applicable/relevant for your study

Not applicable since this is a Study Protocol so only the number of expected participants is reported.

## 16-ii) Primary analysis should be intent-to-treat

Primary analysis should be intent-to-treat, secondary analyses could include comparing only “users”, with the appropriate caveats that this is no longer a randomized sample (see 18-i).

| | 1 | 2 | 3 | 4 | 5 | |
|---|---|---|---|---|---|---|
| subitem not at all important | ◉ | ○ | ○ | ○ | ○ | essential |

## Does your paper address subitem 16-ii?

Copy and paste relevant sections from the manuscript (include quotes in quotation marks "like this" to indicate direct quotes from your manuscript), or elaborate on this item by providing additional information not in the ms, or briefly explain why the item is not applicable/relevant for your study

Tu respuesta

## 17a) For each primary and secondary outcome, results for each group, and the estimated effect size and its precision (such as 95% confidence interval)

## Does your paper address CONSORT subitem 17a? *

Copy and paste relevant sections from the manuscript (include quotes in quotation marks "like this" to indicate direct quotes from your manuscript), or elaborate on this item by providing additional information not in the ms, or briefly explain why the item is not applicable/relevant for your study

Yes. "The statistical analysis planned a priori will consist of a descriptive analysis of the demographic and clinical characteristics of the patients. For the quantitative variables, the mean, standard deviation, 95% confidence intervals (95% CI), variance, standard error, 5% trimmed mean, median, minimum, and maximum will be calculated."

## 17a-i) Presentation of process outcomes such as metrics of use and intensity of use

In addition to primary/secondary (clinical) outcomes, the presentation of process outcomes such as metrics of use and intensity of use (dose, exposure) and their operational definitions is critical. This does not only refer to metrics of attrition (13-b) (often a binary variable), but also to more continuous exposure metrics such as “average session length”. These must be accompanied by a technical description how a metric like a “session” is defined (e.g., timeout after idle time) [1] (report under item 6a).

| | 1 | 2 | 3 | 4 | 5 | |
|---|---|---|---|---|---|---|
| subitem not at all important | ○ | ○ | ◉ | ○ | ○ | essential |

## Does your paper address subitem 17a-i?

Copy and paste relevant sections from the manuscript (include quotes in quotation marks "like this" to indicate direct quotes from your manuscript), or elaborate on this item by providing additional information not in the ms, or briefly explain why the item is not applicable/relevant for your study

Continuous exposure metrics are not as important in the present study as other metrics. Due to Listeo+ mobile app is not designed to keep a constant patient's engagement, the way we are going to measure this sort of secondary process outcomes is through their compliance with the recommendations and their awareness of the critical ones.

## 17b) For binary outcomes, presentation of both absolute and relative effect sizes is recommended

## Does your paper address CONSORT subitem 17b? *

Copy and paste relevant sections from the manuscript (include quotes in quotation marks "like this" to indicate direct quotes from your manuscript), or elaborate on this item by providing additional information not in the ms, or briefly explain why the item is not applicable/relevant for your study

Yes. Descriptive statistics and Multivariate analysis are taken into account.

## 18) Results of any other analyses performed, including subgroup analyses and adjusted analyses, distinguishing pre-specified from exploratory

### Does your paper address CONSORT subitem 18? *

Copy and paste relevant sections from the manuscript (include quotes in quotation marks "like this" to indicate direct quotes from your manuscript), or elaborate on this item by providing additional information not in the ms, or briefly explain why the item is not applicable/relevant for your study

Yes. "For the qualitative variables, frequency distributions with their respective percentages will be calculated. To determine whether there are differences in the level of compliance with surgical recommendations between the group with written recommendations and the group with written recommendations plus the app, Fisher's exact test will be carried out."

### 18-i) Subgroup analysis of comparing only users

A subgroup analysis of comparing only users is not uncommon in ehealth trials, but if done, it must be stressed that this is a self-selected sample and no longer an unbiased sample from a randomized trial (see 16-iii).

| | 1 | 2 | 3 | 4 | 5 | |
|---|---|---|---|---|---|---|
| subitem not at all important | ○ | ○ | ○ | ○ | ◉ | essential |

### Does your paper address subitem 18-i?

Copy and paste relevant sections from the manuscript (include quotes in quotation marks "like this" to indicate direct quotes from your manuscript), or elaborate on this item by providing additional information not in the ms, or briefly explain why the item is not applicable/relevant for your study

Yes. "To assess the influence of sociodemographic and clinical characteristics of the patients in the level of compliance with preoperative recommendations a multivariate logistic regression will be performed. "

## 19) All important harms or unintended effects in each group

(for specific guidance see CONSORT for harms)

## Does your paper address CONSORT subitem 19? *

Copy and paste relevant sections from the manuscript (include quotes in quotation marks "like this" to indicate direct quotes from your manuscript), or elaborate on this item by providing additional information not in the ms, or briefly explain why the item is not applicable/relevant for your study

Not applicable since this is a Study Protocol

## 19-i) Include privacy breaches, technical problems

Include privacy breaches, technical problems. This does not only include physical "harm" to participants, but also incidents such as perceived or real privacy breaches [1], technical problems, and other unexpected/unintended incidents. "Unintended effects" also includes unintended positive effects [2].

| | 1 | 2 | 3 | 4 | 5 | |
|---|---|---|---|---|---|---|
| subitem not at all important | ○ | ○ | ○ | ● | ○ | essential |

## Does your paper address subitem 19-i?

Copy and paste relevant sections from the manuscript (include quotes in quotation marks "like this" to indicate direct quotes from your manuscript), or elaborate on this item by providing additional information not in the ms, or briefly explain why the item is not applicable/relevant for your study

Yes. "Data confidentiality will be protected under the Spanish law that ensures the protection of personal data (Organic Law on Protection of Personal Data, 15/1999, December 13). The researcher of each center will be responsible for keeping a study file containing patient identification and information, including the informed consent form signed by the patients. Throughout the study, all related documents will be located in a secure area of the participating center. Any analysis derived from the study will be performed from an anonymized database; it will not contain any identification of the patient or caregiver but only a numeric code by which it will not be possible to reveal their identities. At the end of the study, the researcher will be responsible for preserving the necessary documentation for at least five years."

## 19-ii) Include qualitative feedback from participants or observations from staff/researchers

Include qualitative feedback from participants or observations from staff/researchers, if available, on strengths and shortcomings of the application, especially if they point to unintended/unexpected effects or uses. This includes (if available) reasons for why people did or did not use the application as intended by the developers.

| | 1 | 2 | 3 | 4 | 5 | |
|---|---|---|---|---|---|---|
| subitem not at all important | ○ | ○ | ○ | ○ | ● | essential |

## Does your paper address subitem 19-ii?

Copy and paste relevant sections from the manuscript (include quotes in quotation marks "like this" to indicate direct quotes from your manuscript), or elaborate on this item by providing additional information not in the ms, or briefly explain why the item is not applicable/relevant for your study

Yes. "Four focus groups –6 to 8 participants each-, will be organized to collect qualitative data about the utility perceived by health professionals as well as intervention group patients experience with the app.
Both research tools will provide very insightful information that will eventually lead to a changes in the Listeo+ functionality or content. "

# DISCUSSION

## 22) Interpretation consistent with results, balancing benefits and harms, and considering other relevant evidence

NPT: In addition, take into account the choice of the comparator, lack of or partial blinding, and unequal expertise of care providers or centers in each group

## 22-i) Restate study questions and summarize the answers suggested by the data, starting with primary outcomes and process outcomes (use)

Restate study questions and summarize the answers suggested by the data, starting with primary outcomes and process outcomes (use).

| | 1 | 2 | 3 | 4 | 5 | |
|---|---|---|---|---|---|---|
| subitem not at all important | ○ | ○ | ◉ | ○ | ○ | essential |

## Does your paper address subitem 22-i? *

Copy and paste relevant sections from the manuscript (include quotes in quotation marks "like this" to indicate direct quotes from your manuscript), or elaborate on this item by providing additional information not in the ms, or briefly explain why the item is not applicable/relevant for your study

Not applicable since this is a Study Protocol

## 22-ii) Highlight unanswered new questions, suggest future research

Highlight unanswered new questions, suggest future research.

| | 1 | 2 | 3 | 4 | 5 | |
|---|---|---|---|---|---|---|
| subitem not at all important | ○ | ◉ | ○ | ○ | ○ | essential |

## Does your paper address subitem 22-ii?

Copy and paste relevant sections from the manuscript (include quotes in quotation marks "like this" to indicate direct quotes from your manuscript), or elaborate on this item by providing additional information not in the ms, or briefly explain why the item is not applicable/relevant for your study

Not applicable since this is a Study Protocol

## 20) Trial limitations, addressing sources of potential bias, imprecision, and, if relevant, multiplicity of analyses

## 20-i) Typical limitations in ehealth trials

Typical limitations in ehealth trials: Participants in ehealth trials are rarely blinded. Ehealth trials often look at a multiplicity of outcomes, increasing risk for a Type I error. Discuss biases due to non-use of the intervention/usability issues, biases through informed consent procedures, unexpected events.

| | 1 | 2 | 3 | 4 | 5 | |
|---|---|---|---|---|---|---|
| subitem not at all important | ○ | ○ | ○ | ○ | ◉ | essential |

## Does your paper address subitem 20-i? *

Copy and paste relevant sections from the manuscript (include quotes in quotation marks "like this" to indicate direct quotes from your manuscript), or elaborate on this item by providing additional information not in the ms, or briefly explain why the item is not applicable/relevant for your study

Yes. "Limitations
The present study has some limitations related to the design of the intervention and the methodology used. First, it is a randomized clinical trial with an open-label design. Although not blinding patients and professionals could lead to potential bias in the interpretation of results, this type of design is widely accepted in complex non-pharmacological interventions (e.g., surgery and medical devices) [44] in which masking cannot be applied. Second, Listeo+ has been evaluated as a complement to SOC (written recommendations). In this sense, other published clinical trials based on MAS and Patient Support Programs also use this methodological approach where intervention is assumed as a complement to SOC [45-46]."

## 21) Generalisability (external validity, applicability) of the trial findings

NPT: External validity of the trial findings according to the intervention, comparators, patients, and care providers or centers involved in the trial

## 21-i) Generalizability to other populations

Generalizability to other populations: In particular, discuss generalizability to a general Internet population, outside of a RCT setting, and general patient population, including applicability of the study results for other organizations

| | 1 | 2 | 3 | 4 | 5 | |
|---|---|---|---|---|---|---|
| subitem not at all important | ○ | ○ | ○ | ○ | ◉ | essential |

## Does your paper address subitem 21-i?

Copy and paste relevant sections from the manuscript (include quotes in quotation marks "like this" to indicate direct quotes from your manuscript), or elaborate on this item by providing additional information not in the ms, or briefly explain why the item is not applicable/relevant for your study

Yes. "Furthermore, the interest of the study lies in the increase in MAS and the adoption of mobile devices and acceptance of mobile Health Apps by the population. Thus, the number of MAS operations in developed countries has continued to increase in recent years. In 2015 in Spain, 1,632,824 MAS operations were performed, corresponding to an increase of 4.2% from the period of 2010-2015 [11]"

## 21-ii) Discuss if there were elements in the RCT that would be different in a routine application setting

Discuss if there were elements in the RCT that would be different in a routine application setting (e.g., prompts/reminders, more human involvement, training sessions or other co-interventions) and what impact the omission of these elements could have on use, adoption, or outcomes if the intervention is applied outside of a RCT setting.

| | 1 | 2 | 3 | 4 | 5 | |
|---|---|---|---|---|---|---|
| subitem not at all important | ○ | ○ | ○ | ○ | ● | essential |

## Does your paper address subitem 21-ii?

Copy and paste relevant sections from the manuscript (include quotes in quotation marks "like this" to indicate direct quotes from your manuscript), or elaborate on this item by providing additional information not in the ms, or briefly explain why the item is not applicable/relevant for your study

Yes. "It also highlights that the penetration level of smartphones is also increasing, even among the elderly, reducing the generation gap [42]. Finally, data from a local survey on the use of mobile Health Apps shows that 73.8% of patients would use them if recommended by their doctor, which suggests a high level of acceptance of mobile Health Apps by the population [43]."

## OTHER INFORMATION

## 23) Registration number and name of trial registry

## Does your paper address CONSORT subitem 23? *

Copy and paste relevant sections from the manuscript (include quotes in quotation marks "like this" to indicate direct quotes from your manuscript), or elaborate on this item by providing additional information not in the ms, or briefly explain why the item is not applicable/relevant for your study

The Study has a Andalusian Regional Ministry of Health Registration number (0032-N-16) and will be included in the presentation letter as it was suggested by the editorial team.

## 24) Where the full trial protocol can be accessed, if available

### Does your paper address CONSORT subitem 24? *

Cite a Multimedia Appendix, other reference, or copy and paste relevant sections from the manuscript (include quotes in quotation marks "like this" to indicate direct quotes from your manuscript), or elaborate on this item by providing additional information not in the ms, or briefly explain why the item is not applicable/relevant for your study

The trial protocol is not available so far, but we're planning to publish it in our website once this research study protocol is published in JMIR study protocols

## 25) Sources of funding and other support (such as supply of drugs), role of funders

### Does your paper address CONSORT subitem 25? *

Copy and paste relevant sections from the manuscript (include quotes in quotation marks "like this" to indicate direct quotes from your manuscript), or elaborate on this item by providing additional information not in the ms, or briefly explain why the item is not applicable/relevant for your study

Yes. "Acknowledgements
This project has received funding from the public Institute of Health Carlos III (ISCIII), which is part of the Ministry of Economy and Competitiveness (MEC) of Spain (file number: DTS15/00228), and the Foundation for Healthcare and Progress (file number: PI-0447-2014) which is part of the Health Department of the Regional Government of Andalusia (Spain)."

## X27) Conflicts of Interest (not a CONSORT item)

### X27-i) State the relation of the study team towards the system being evaluated

In addition to the usual declaration of interests (financial or otherwise), also state the relation of the study team towards the system being evaluated, i.e., state if the authors/evaluators are distinct from or identical with the developers/sponsors of the intervention.

| | 1 | 2 | 3 | 4 | 5 | |
|---|---|---|---|---|---|---|
| subitem not at all important | ○ | ○ | ○ | ○ | ○ | essential |

## Does your paper address subitem X27-i?

Copy and paste relevant sections from the manuscript (include quotes in quotation marks "like this" to indicate direct quotes from your manuscript), or elaborate on this item by providing additional information not in the ms, or briefly explain why the item is not applicable/relevant for your study

Yes. "Conflicts of Interest
The authors actively participated in the design of the intervention (Listeo+ mobile health app) but have no conflict of interest affecting finances due to the app copyright belongs to the Andalusian Regional Healthcare Ministry."

## About the CONSORT EHEALTH checklist

## As a result of using this checklist, did you make changes in your manuscript? *

- ○ yes, major changes
- ◉ yes, minor changes
- ○ no

## What were the most important changes you made as a result of using this checklist?

Conflict of interests and some minor changes when the qualitative data gathering is reported.

## How much time did you spend on going through the checklist INCLUDING making changes in your manuscript *

Around 10 hours.

## As a result of using this checklist, do you think your manuscript has improved? *

- ◉ yes
- ○ no
- ○ Otros:

## Would you like to become involved in the CONSORT EHEALTH group?

This would involve for example becoming involved in participating in a workshop and writing an "Explanation and Elaboration" document

- (•) yes
- ( ) no
- ( ) Otros:

## Any other comments or questions on CONSORT EHEALTH

Tu respuesta

## STOP - Save this form as PDF before you click submit

To generate a record that you filled in this form, we recommend to generate a PDF of this page (on a Mac, simply select "print" and then select "print as PDF") before you submit it.

When you submit your (revised) paper to JMIR, please upload the PDF as supplementary file.

Don't worry if some text in the textboxes is cut off, as we still have the complete information in our database. Thank you!

## Final step: Click submit !

Click submit so we have your answers in our database!

ENVIAR

Nunca envíes contraseñas a través de Formularios de Google.

Google no creó ni aprobó este contenido. Denunciar abuso - Condiciones del servicio - Condiciones adicionales

Google Formularios
