# Supplementary material for: Effect of a Mobile App on Preoperative Patient Preparation for Major Ambulatory Surgery: Protocol for a Randomized Controlled Trial
Source: JMIR Res Protoc. 2019 Jan 16;8(1):e10938. doi: 10.2196/10938 (PMC6352007; doi:10.2196/10938)
Supplement: Multimedia Appendix 4 [file resprot_v8i1e10938_app4.pdf]

Informe de evaluación: Proyectos de Investigacion en Salud (AES 2015). Modalidad Proyectos de Desarrollo Tecnológico en salud.

EXPEDIENTE:	DTS15/00228
INVESTIGADOR PRINCIPAL:	SANTANA LOPEZ, VICENTE
CENTRO DE REALIZACIÓN:	FUNDACION AGENCIA DE CALIDAD SANITARIA DE ANDALUCIA
TÍTULO:	Impacto de la app Listeo+ sobre la reducción de cancelaciones de intervenciones quirúrgicas, mejora de la seguridad del paciente y aceptabilidad por parte de profesionales y pacientes

a) Valoración del equipo de investigación (0-35)	29,00
b) Valoración del proyecto (0-65)	56,00
PUNTUACIÓN TOTAL:	85,00

Proyecto de implantación-validación multicéntrica de una App, en Sistema Andaluz de Salud, dirigido a la preparación prequirúrgica de los pacientes con el objeto de reducir las tasas de cancelación en cirugía programada. Parte de la base del uso de las nuevas tecnologías por los pacientes. El equipo investigador es esencialmente gestor y tecnólogo, con poca experiencia investigadora. El proyecto es claro con objetivos, recursos y potenciales resultados muy bien definidos. Quedan sin definir aspectos de potencial transferencia al mercado.
