# Supplementary material for: Effect of a Mobile App on Preoperative Patient Preparation for Major Ambulatory Surgery: Protocol for a Randomized Controlled Trial
Source: JMIR Res Protoc. 2019 Jan 16;8(1):e10938. doi: 10.2196/10938 (PMC6352007; doi:10.2196/10938)
Supplement: Multimedia Appendix 5 [file resprot_v8i1e10938_app5.pdf]

INFORME FINAL DE EVALUACIÓN
FPS 2014 Proyectos de Investigación - Proyectos Investigación

**Modalidad**	Proyectos de Investigación en Salud: Temática General.		
**Expediente**	PI-0447-2014		
**Titulo del proyecto**	Impacto de la App Listeo+ Sobre la Reducción de Cancelaciones de Intervenciones Quirúrgicas y Grado de Aceptabilidad Por Parte de Pacientes y Profesionales		
**Investigador/a principal**	Manuel Herrera-Usagre		
**Invest. Colaboradores/as**	Pastora Pérez Pérez, Marta Vázquez , Antonio Torres Olivera, Javier Ferrero Álvarez-Rementería, Manuel Enrique Pacheco Mera, Susana Cruces Roldán, Vicente Santana López, MIGUEL PORRAS POVEDANO, JUAN JAVIER CEREZO ESPINOSA MONTEROS		
**Entidad beneficiaria**	Fundacion Pública Andaluza Progreso y Salud		
**Centro/s**	H. Riotinto (A.G.S. Norte de Huelva), Hospital de Alta Resolución de Utrera, ACSA-Agencia de Calidad Sanitaria de Andalucía		
**Puntuación del proyecto**	**70,2**	**Punto de corte**	70
**Estado del Proyecto**	**Seleccionado para financiar**		

Una vez finalizada la fase de evaluación, siguiendo los criterios recogidos en el apartado 5 de la Convocatoria de ayudas para la financiación de proyectos de investigación Biomédica y en Ciencias de la Salud en Andalucía para el año 2014, **los resultados de la evaluación del proyecto** indicado anteriormente son los siguientes:

	**Criterios de Evaluación**	**Puntuación del Proyecto**	**Puntuación Máxima posible**	**Comparación con Puntuación Media de todos los Proyectos (1)**
**Criterios Científicos**	1. Novedad y relevancia (2)	APTO	APTO	
	2. Calidad científico-técnica y viabilidad	40,5	45	+
	3. Equipo investigador	16,5	20	+
	4. Aplicabilidad de los resultados	7	10	+
	**Total evaluación científica**	**64**	**75**	**+**
**Líneas estratégicas(3)**	1. Investigadores emergentes	0	8	
	2. Terapias Avanzadas	0	3	
	3. Estudio de intervención	1,2	3	
	4. Actividades con Empresas	0	3	
	5. Registro Propiedad intelectual o industrial	2	2	
	6. Evaluación EXPOST	0	2	
	7. Grupos Multidisciplinares, interniveles e intercentros	3	3	
	8. Perspectiva de género	0	2	
	9. Equidistribución Geográfica	0	1,5	
	10. Colectivo	0	8	
	**Total Líneas estratégicas**	**6,2**	**25**	
	**TOTAL DEL PROYECTO**	**70,2**	**100**	**+**

(1) +: Puntuación superior a la media; -: Puntuación inferior a la media

(2) Según el apartado cinco de la convocatoria, para la evaluación del resto de criterios es necesario obtener una calificación de APTO en este criterio

(3) Sólo para proyectos cuya suma de los puntos de los criterios científicos 2 y 4 es al menos 39.2 puntos.

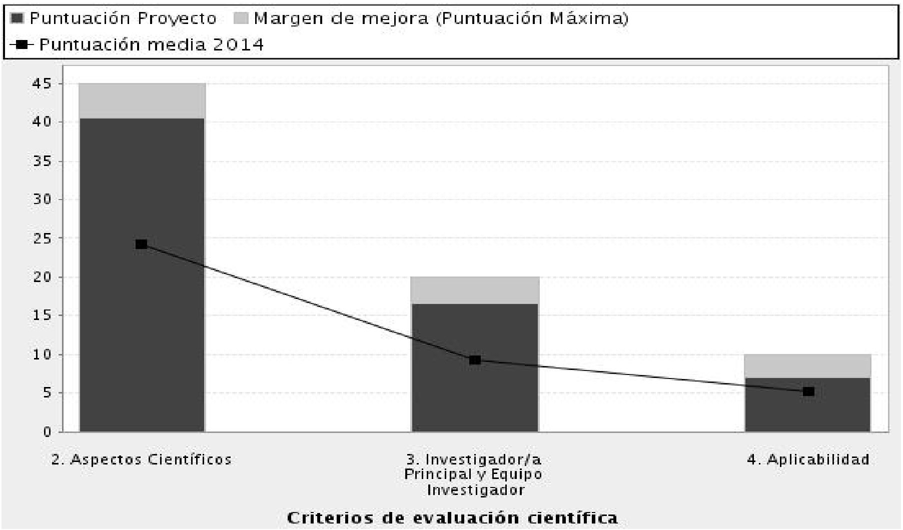

Figura 1. Resultados comparados de la evaluación científica del proyecto por Criterios.

1. COMENTARIOS ACERCA DE LA EVALUACIÓN CIENTÍFICA

Criterio 1: Novedad y relevancia de la propuesta. APTO

Criterio 2: Calidad científico-ténica y viabilidad del proyecto (40.5/45.0)

El proyecto persigue evaluar la eficacia y usabilidad de la aplicación Listeo+ en la reducción del número de cancelaciones quirúrgicas, Identificando las causas que se asocian al no cumplimiento de las recomendaciones por los pacientes, cuyo resultado es la cancelación quirúrgica. Pretende analizar la satisfacción de los pacientes y la utilidad percibida de los profesionales sanitarios respecto a la implantación de la aplicación Listeo+, y al tiempo analizar el impacto económico de la intervención mediante un análisis de costes. Todo ello en el marco de un estudio experimental prospectivo. La investigación es clara, concisa y se apoya en literatura actualizada. Las hipótesis son plausibles y contrastables. Los objetivos concretos, medibles y alcanzables. La metodología es adecuada a los objetivos, excepto en lo relativo al objetivo quinto.

Las limitaciones aunque están discutidas, no lo están con demasiada profundidad. El plan de trabajo está explicitado tanto en el tiempo como para los investigadores aunque podría mejorar el detalle de las responsabilidades concretas de cada uno de ellos.

Criterio 3: Equipo Investigador. (16.5/20.0)

Euqipo con capacidad y experiencia para llevar a cabo este estudio. Experiencia en proyectos competitivos y con publicaciones en revista de impacto.

Criterio 4: Aplicabilidad del proyecto para el Sistema Sanitario Público Andaluz (7.0/10.0)

El estudio es viable y se aportan pistas que hacen creer que se puede cumplir el calendario. Tanto el plan de difusión como la divulgación de los avances de la investigación están contemplados con detalle en la memoria de investigación.

2. COMENTARIOS ACERCA DE LA EVALUACIÓN DE LA INVESTIGACIÓN CONJUNTA CON EMPRESAS Y/O PROTECCIÓN DEL CONOCIMIENTO

Investigación conjunta con empresas:

Protección del conocimiento: Se prevé que el presente proyecto pueda generar resultados susceptibles de registro en la propiedad industrial o intelectual distintos de los derivados de publicaciones cientificas. En particular, una aplicación informática para movil que sirva de ayuda y recordatorio de todas las pautas y comprobaciones que se deben seguir antes de someterse a una intervención quirúrgica.

La protección del conocimiento generado en los proyectos de I+i constituye un pilar básico en el proceso de valorización y transferencia de resultados de investigación, cuyo objetivo último es garantizar el traslado eficiente y la aplicación de dichos resultados en Salud. Si desea asesoramiento o tiene alguna cuestión relacionada con las colaboraciones con empresas y/o asuntos de propiedad industrial o intelectual, puede ponserse en contacto pon los técnicos de la OTT por teléfono (955040450) y correo electrónico (ott.sspa@juntadeandalucia.es).

3. INFORMACIÓN SOBRE OTRAS OPORTUNIDADES DE FINANCIACIÓN Y LOS SERVICIOS QUE PROPORCIONA LA RED DE FUNDACIONES GESTORAS DE INVESTIGACIÓN DEL SSPA (RFGI-SSPA)

Si desea información sobre **otras oportunidades de financiación** tiene a su disposición a través del Portal de Servicios de Gestión y Apoyo a la I+i del Sistema Sanitario Público de Andalucía **INVESTIGA+** las siguientes herramientas:

- Buscador de convocatorias: Encuentra según el tipo de ayuda, ámbito, entidad financiadora, estado o por el nombre, la convocatoria que mejor se ajusta a las necesidades de tu actividad. Acceda

- Agenda de Oportunidades de Financiación para la I+i Biomédica, (formato calendario imprimible en A4). Instrumento de planificación y consulta en el que se incluyen los **requisitos** imprescindibles para la participación en las convocatorias, y un cronograma con los plazos efectivos o previstos para la presentación de solicitudes, y con un **hipervínculo a la ficha detallada** de cada una de las ayudas.
Este año, como novedad, se edita únicamente en formato digital, lo que permite una actualización diaria de las ayudas. Consúltela

Además en **INVESTIGA+** , podrá encontrar toda la información correspondiente a la cartera de servicios que ofrece la Red de Fundaciones Gestoras de la Investigación del Sistema Sanitario Público de Andalucía (RFGI-SSPA). La RFGI-SSPA ofrece apoyo, soporte y personalidad jurídica según su ámbito geográfico. Está compuesta por siete fundaciones: FCÁDIZ (Cádiz), FIBICO (Córdoba), FABIS (Huelva), FIMABIS (Málaga), FISEVI (Sevilla), FIBAO (con carácter interprovincial, dando cobertura a Granada, Jaén y Almería) y la Fundación Pública Andaluza Progreso y Salud (FPS) como entidad coordinadora de la Red.

La **Cartera de Servicios de Apoyo a la I+i** que la RFGI-SSPA ofrece al Investigador/a es la siguiente:

1. Asesoramiento para la captación de financiación

- Difusión de oportunidades de financiación, de búsquedas de socios, de oportunidades de colaboración con empresas (demandas tecnológicas, colaboración público-privada)
- Formación para la planificación de la captación de recursos

2. Gestión de ayudas

- Preparación y seguimiento de las propuestas
- Gestión económica de las ayudas y justificación

3. Gestión de ensayos clínicos y estudios observacionales

- Difusión de oportunidades de investigación clínica y gestión administrativa de ensayos clínicos
- Asesoramiento para la investigación clínica independiente

4. Asesoramiento de Proyectos Internacionales

- Preparación de propuestas y búsquedas de socios
- Formación / Información

5. Apoyo Metodológico y Estadístico

- Formación en búsquedas (BV-SSPA + OTT-SSPA)
- Asesoramiento metodológico (proyectos y tesis doctorales)
- Análisis estadístico y diseño de bases de datos

6. Asesoramiento y gestión de la protección y transferencia de los resultados

- Asesoramiento para las publicaciones y servicios de traducción
- Acuerdos de colaboración público-privada
- Asesoramiento y gestión para la protección y transferencia de resultados
